# Natural Products as Modulators of the DNA Damage Response and Oncogenic Signaling in Breast Cancer Therapy

**DOI:** 10.3390/ijms27167107

**Published:** 2026-08-08

**Authors:** Maria Cuomo, Francesco Errichiello, Carolina Di Meo, Martino Forino, Luigi Frusciante, Michelino De Laurentiis, Antonio Giordano, Luigi Alfano

**Affiliations:** 1 Department of Medical Biotechnologies, University of Siena, 53100 Siena, Italy; m.cuomo@student.unisi.it; 2 Department of Agricultural Sciences, Grape and Wine Science Division, University of Napoli Federico II, 83100 Avellino, Italy; francesco.errichiello@unina.it (F.E.); forino@unina.it (M.F.); fruscian@unina.it (L.F.); 3 Department of Breast and Thoracic Oncology, Istituto Nazionale Tumori-IRCCS-Fondazione G. Pascale, 80131 Napoli, Italy; caro.dimeo@studenti.unina.it (C.D.M.); m.delaurentiis@istitutotumori.na.it (M.D.L.); 4 Sbarro Institute for Cancer Research and Molecular Medicine, Center for Biotechnology, College of Science and Technology, Temple University, Philadelphia, PA 19122, USA

**Keywords:** natural products, DNA damage response, homologous recombination, breast cancer, triple-negative breast cancer, chemosensitization, polyphenols, terpenoids, alkaloids, PARP inhibitors

## Abstract

Breast cancer (BC) is the most frequently diagnosed malignancy and generally the leading cause of cancer-related mortality among women worldwide. Molecular targeted therapies, including endocrine treatment for hormone-responsive tumors, anti-HER2 agents for HER2-positive tumors and PARP inhibitors (PARPis) for homologous recombination-deficient (HRD) tumors, have significantly improved patient outcomes, but several clinical challenges are still open. The development of acquired resistance strongly limits the long-term efficacy of current treatment strategies, underscoring the necessity to identify novel therapeutic approaches for breast cancer therapy. In this review, we summarize and discuss recently investigated natural compounds with anti-breast cancer activity, ranging from polyphenols, terpenoids, alkaloids, sulfur-containing compounds and the emerging plant-derived extracellular vesicles. We focus on their ability to induce DNA damage or oxidative stress, modulating the DNA damage response (DDR) and interfering with key oncogenic signaling pathways. We also discuss the potential of these compounds to enhance the efficacy of conventional therapies, thereby offering a promising role in overcoming clinical resistance. Despite clinical relevance remains under development and further studies are required; many of the natural compounds examined here appear to effectively target DDR signaling and oncogenic pathways, supporting their potential use in breast cancer therapy.

## 1. Introduction

Breast cancer stands as a leading cancer-related cause of death and illness across the world, affecting about 30% of all female cancer cases [1]. The development of this condition is due to multiple factors, which combine genetic predispositions with environmental elements such as age, hormones, lifestyle, nutrition, and social factors. The process of acquiring essential gene mutations, which control cell growth, DNA repair and cell cycle functions, results from the combination of these factors [2]. The most prevalent cancer predisposition involves sporadic mutation events in the DNA, but a small portion of cancers show a hereditary form, as in breast cancers, where the mutations of *BRCA1* and *BRCA2* genes were the most prevalent and transmitted to the progeny [2]. Breast cancer exists as a heterogeneous group of diseases according to different biological characteristics. It consists of distinct molecular subtypes that show different clinical behavior, outcome and response to treatment. The molecular classification of breast cancer depends on the presence of three specific receptors, which include the estrogen receptor (ER), the progesterone receptor (PR), and human epidermal growth factor receptor 2 (HER2) [3]. Based on the receptors expression, four tumor types were defined: luminal A; luminal B (with low or high proliferative activity); HER2-positive tumors; and triple-negative breast cancer (TNBC), which lacks ER, PR and HER2 expression [3]. TNBC represents 10–15% of the invasive breast cancer cases, and it is considered one of the most aggressive subtypes, because it grows rapidly, spreads quickly and shows a poor treatment response [1]. The heterogeneity of TNBC has become clearer through genomic and transcriptomic analysis, which has led to the identification of four distinct subgroups: basal-like, mesenchymal, immunomodulatory and androgen receptor-positive [1]. The evaluation of receptors and molecular markers is now a key part of clinical strategies to make therapeutic decisions; it defines personalized prevention and surveillance strategies. The treatment of breast cancer has made major progress over the last few decades since medicine moved from general therapeutic methods to more accurate and personalized treatment strategies, as discussed later. However, the identification of new molecules to be employed for cancer therapy is still necessary to increase the efficacy of tumor treatment and to bypass the development of acquired resistance mechanisms. Recently, the investigation of natural compounds, as a source of undiscovered molecules for the development of new drugs or as a scaffold for already used therapies, has attracted increased interest across the scientific community.

## 2. Advances in Breast Cancer Therapy

**Figure 1:**
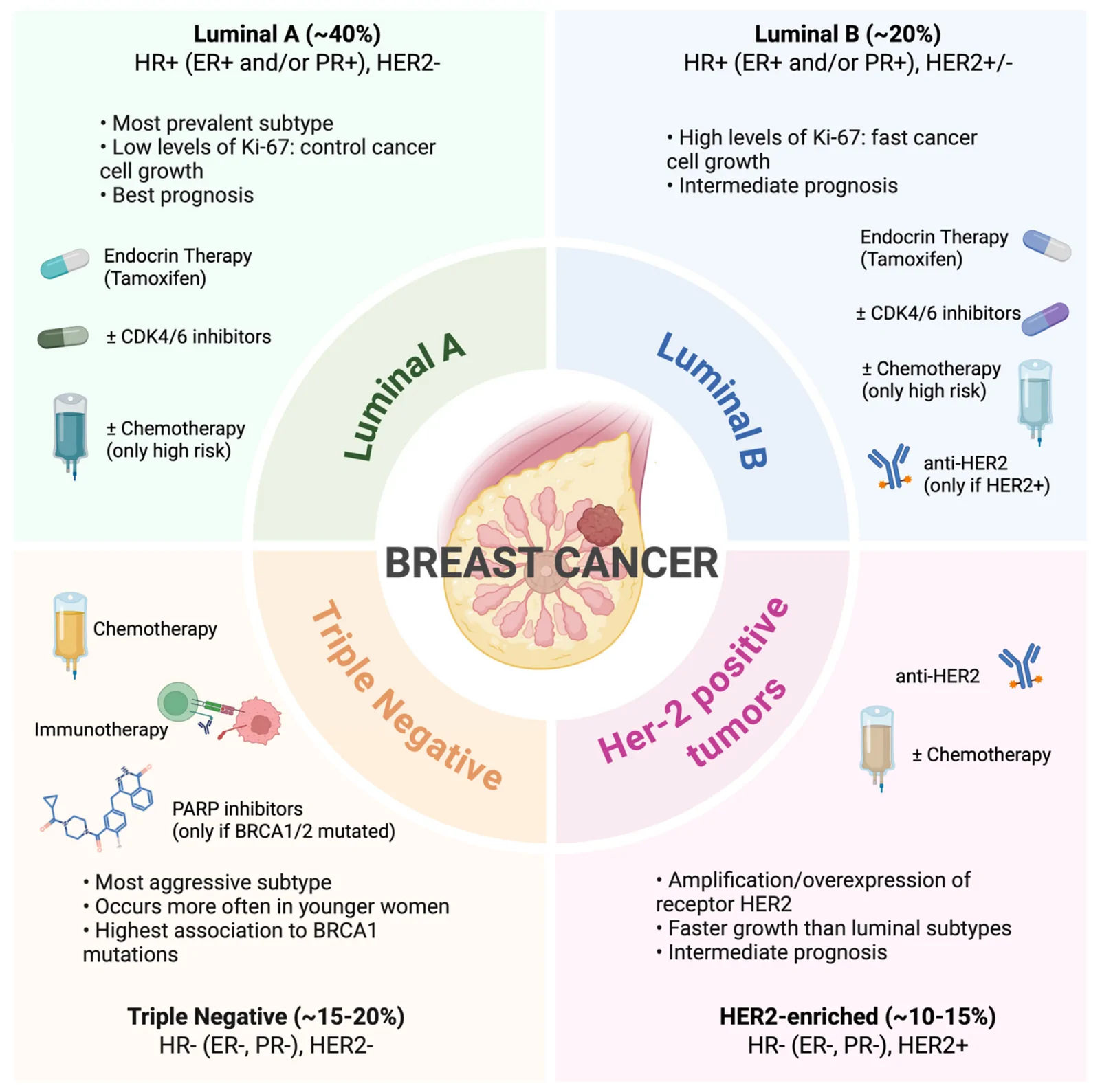
Breast cancer molecular subtypes and current therapeutic strategies. Schematic overview of the four major molecular subtypes of breast cancer, classified according to hormone receptor and HER2 expression status. The figure highlights the distinct biological features of each subtype and summarizes the principal therapeutic approaches currently adopted in clinical practice, including endocrine therapy (for example, Tamoxifen), anti-HER2 target therapy, chemotherapy, immunotherapy, and CDK4/6 inhibitors. Created in BioRender. De Laurentiis, M. (2026) https://BioRender.com/3nxk2qy.

Surgery, radiotherapy and chemotherapy have been the pillars of BC therapy, bringing about a significant reduction in mortality rates but with reduced cancer cell specificity, as well [4]. The advent of molecular therapy targeting specific molecular vulnerabilities of cancer cells has significantly increased the success of cancer treatment, reducing healthy tissue toxicity (Figure 1). Among these, the endocrine therapies, such as selective estrogen receptor degraders (SERDs), selective estrogen receptor modulators (SERMs) and aromatase inhibitors, have been a milestone of great importance for hormone-sensitive tumors by showing benefits for survival extension and reduction in recurrence risk. Tamoxifen, the first-in-class SERM, has long represented the gold standard of endocrine therapy and remains one of the most used treatments for HR+ tumors [5]. Moreover, Fulvestrant, a SERD that also acts as a selective ER inhibitor, represents the gold standard for advanced HR+ breast cancer [6]. Resistance to endocrine therapy is frequently observed, especially in advanced disease, and it is usually associated with mutations in the estrogen receptor 1 (*ESR1*) gene; the use of Lasofoxifene, a third-generation SERM [7], is currently under clinical investigation in *ESR1*-mutant BC. Recently, the introduction of the cyclin-dependent kinase 4/6 (CDK4/6) inhibitors (e.g., abemaciclib, ribociclib, and palbociclib) has significantly improved the management of luminal BC when used combined to endocrine therapy, establishing this combination as the standard first-line treatment for advanced HR+ disease. Ongoing clinical trials are evaluating the combination of lasofoxifene with abemaciclib in patients harboring *ESR1*-mutations [8]. Many other pathways play a crucial role in endocrine resistance, such as the activation of the phosphoinositide 3-kinase (PI3K)/protein kinase B (AKT)/mammalian target of rapamycin (mTOR) pathway; this observation has led to the Food and Drug Administration (FDA) approval of Capivasertib as the first AKT inhibitor for hormone receptor positive (HR+) advanced breast cancer [9]. Similarly, advances in the understanding of HER2 signaling have led to the development of highly effective targeted therapies, leading to an improvement of clinical outcomes and prognosis for HER2+ patients. Trastuzumab, the first anti-HER2 targeted monoclonal antibody, revolutionized the therapeutic response and paved the way for the development of additional anti-HER2 agents, including Pertuzumab and the more sophisticated antibody–drug conjugates (ADCs), such as Trastuzumab–emtansine (T-DM1) and Trastuzumab–deruxtecan (T-DXd), which subsequently prolonged the progression-free survival (PFS) and overall survival (OS) for these patients [4,10]. Despite these remarkable advances, treatment-related toxicities and acquired resistance of tumors strongly limit the long-term efficiency of these treatments [10]. Increasing evidence indicates that response to therapy is strongly influenced by the tumor microenvironment (TME) which plays a key role in immune evasion and acquisition of therapeutic resistance, through complex interactions between cancer cells and the surrounding stromal and immune cells [11]. Therefore, the TME has emerged as an attractive therapeutic target by using immune checkpoint blockade (ICB) [12]. Two recent clinical trials led to the Food and Drug Administration (FDA) approval of Pembrolizumab, a monoclonal antibody that targets programmed death protein-1 (PD-1) [13], as neoadjuvant immunotherapy for patients with TNBC [14,15]. Although the promising effect of ICBs, the adverse effects, such as off-target toxicity, limit their use as monotherapy [16]. Conversely, ICBs are currently explored in BC therapy in combinational strategies, for example with ADCs in HER2+ breast cancer [17] and TNBC [18]. Interestingly, CDK4/6 inhibitors also exhibit immunomodulatory activities beyond their well-established antiproliferative effects. These agents have been shown to enhance antigen presentation in cancer cells, promoting the infiltration and activity of cytotoxic CD8+ T cells within the TME, providing a strong rationale for their combination with immunotherapy [19]. Among the different BC molecular subtypes, immunotherapy strategies are really promising, especially for TNBC, due to tumor immune infiltration and high levels of immune markers [20]. Recent insights into the molecular heterogeneity of TNBC have significantly expanded the therapeutic options available for this aggressive subtype, which has been the most challenging, due to the lack of actionable therapeutic targets. Approximately 80% of TNBC overexpress the trophoblast cell surface antigen 2 (Trop2), a transmembrane glycoprotein, which has emerged as an attractive therapeutic target in TNBC using ADCs, such as Sacituzumab govitecan (SG), which received FDA approval [21]. Moreover, the PI3K/AKT/mTOR signaling pathway is frequently activated in TNBC, and the addition of Capivasertib to first-line paclitaxel significantly improved both PFS and OS, particularly in tumors harboring *PIK3CA*, *AKT1*, or *PTEN* alterations [22]. Furthermore, mutations in BRCA1 or BRCA2, which led to HRD, are particularly common in TNBC, making these patients eligible for treatment with PARPi, as discussed in the following section. Despite all the remarkable advantages achieved in BC clinical therapy the development of secondary resistance continues to limit progress in cancer treatment, prompting scientists to find new predictive biomarkers for better patient stratification and novel therapeutic agents as well [23,24].

## 3. Targeting DDR in Breast Cancer Therapy

**Figure 2:**
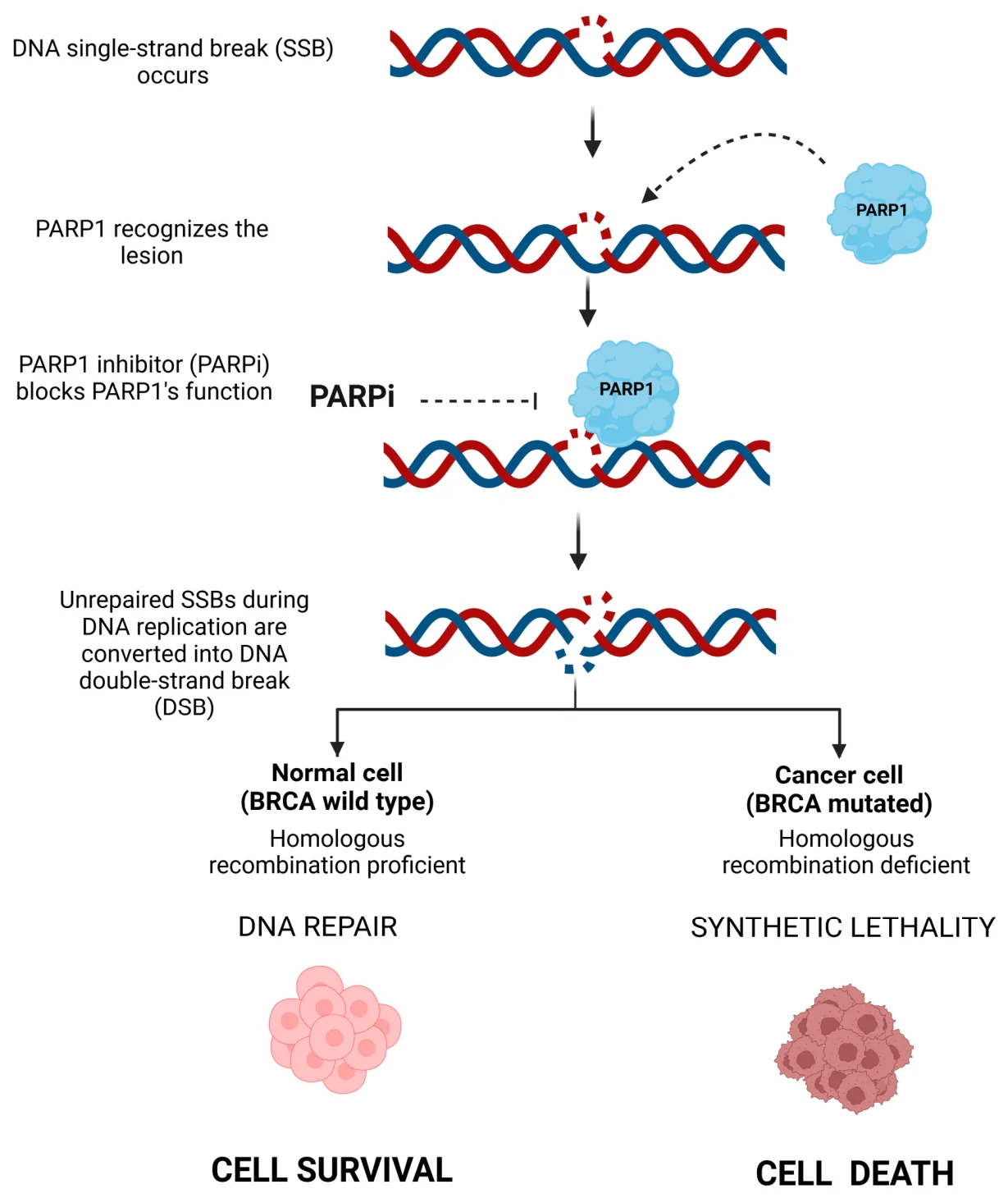
Synthetic lethality induced by PARPi in BRCA-mutated tumors. Poly (ADP-ribose) polymerases (PARPs) play a critical role in the repair of DNA single-strand breaks (SSBs) through the base excision repair (BER) pathway. PARP inhibitors, such as olaparib, block this repair process by trapping PARP enzymes at sites of DNA damage, leading to the accumulation of unrepaired SSBs. During DNA replication, these lesions are converted into double-strand breaks (DSBs). In tumors harboring defects in homologous recombination repair (HRR), such as those carrying BRCA1 or BRCA2 mutations, DSBs cannot be accurately repaired. Consequently, cells rely on the error-prone non-homologous end joining (NHEJ) pathway, resulting in genomic instability and ultimately tumor cell death through synthetic lethality. Created in BioRender. De Laurentiis, M. (2026) https://BioRender.com/w15qix9.

Targeting DDR represents a promising strategy in breast cancer therapy by increasing the PFS and OS upon treatment with DDR inhibitors [25]. Human cells rely on a complex network of DDR pathways to detect and remove DNA lesions for the accurate maintenance of genomic stability. DDR deals with the resolution of DNA damage generated by both exogenous and endogenous insults, as from the normal DNA metabolism or reactive oxygen species (ROS) from cellular metabolism [26]. Several chemical agents used in BC therapy can cause different types of DNA lesions; for example, mitomycin C (MMC) and platinum-based drugs are crosslinking agents inducing covalent links between DNA bases of the same or different DNA strands [27]. Ionizing radiation, derived from radiotherapy, also causes various forms of DNA damage, including oxidation of DNA bases, single-stranded and double-stranded DNA breaks (SSBs and DSBs respectively) [27]. More recently, the advent of ADCs in breast cancer therapy, carrying as payload DNA-damaging agents, has renewed interest in DNA repair mechanisms as targets for cancer therapy [28]. DSBs are the most toxic DNA lesion and can be repaired by two principal mechanisms: homologous recombination (HR) or non-homologous end-joining (NHEJ) [29]. HR can occur only during the S/G2 phases of the cell cycle, when the sister chromatid can provide the sequence information for repairing the damaged DNA, leading to error-free repair. In contrast, NHEJ involves the direct ligation of the two DSB ends and so it is an error-prone process [30]. In cancer cells, some of the DDR pathways are commonly compromised, leading to genomic instability. However, the loss of DDR pathway regulation occurs in cancer cells, making them vulnerable to further DNA damage [31]. The best-known cancer-associated examples of defective components of the HR machinery are the tumor suppressor genes *BRCA1* and *BRCA2*. These two genes, as discussed above, are frequently found mutated in breast cancer as well as in other cancer types, such as ovarian, prostate and pancreatic cancers, defined as HRD-tumors [32]. A cancer cell with a defect in the DDR relies on compensatory pathways for survival, thereby creating a therapeutic opportunity; selectively inhibiting the backup pathway promotes the accumulation of unrepaired DNA damage, ultimately resulting in cell death, a drug strategy known as synthetic lethality (Figure 2) [33]. Translating this concept to DDR therapeutics, one event is genetic (germline or sporadic) and specific to the tumor, while the second loss-of-function event is achieved pharmacologically through treatment with a DDR inhibitor. These observations have led to the approval by the European Medicines Agency (EMA) in 2014 and, one year later, by the FDA of the PARP inhibitor, Olaparib, as the drug of choice for the treatment of patients with HR-deficient tumors [34]. Olaparib and Talazoparib are approved for the treatment of HER2-negative breast cancer carrying *BRCA1* or *BRCA2* mutations [35]. Patients who are treated with PARPi have been shown to acquire resistance to these drugs over time; different mechanisms have been described as responsible for this developed resistance such as reversion of mutation of *BRCA1*/*2* [36], upregulation of drug efflux pumps [37], stabilization of truncated BRCA1 protein [38] and loss of PARP1 [39]. ATRi may also circumvent the restored checkpoint in resistant cells, thereby promoting more frequent collision between replication forks and ssDNA gaps [40]. So, from the advent of the synthetic lethal concept, many new interactions were described for cancer therapy but also in resistance mechanisms. An example came from TNBC, in which about 20% of patients carry *BRCA* mutations, limiting the usage of PARPi [41] but, at the same time, almost 50% of TNBC cases exhibit loss of the G1/S checkpoint. Clinical trials evaluating the combination of Olaparib (PARPi) and Ceralasertib (ATRi) have indicated potential therapeutic advantages in a subset of TNBC patients with no BRCA mutations, who would otherwise be unlike to respond to Olaparib treatment alone [42]. HRD can also result from mutations in other genes involved in the HR pathway, such as *PALB2*, *RAD51C*, *CtIP*, and *BRIP1*, as well as from their epigenetic silencing caused by promoter hypermethylation [43]. Collectively, these alterations give rise to a phenotype known as “*BRCAness*”, which describes tumors that, despite wild-type HR genes, exhibit impaired HR repair and may therefore serve as a predictive biomarker of sensitivity to PARPi [44]. These findings highlight that several DDR components constitute actionable therapeutic targets in breast cancer as well as for other tumors harboring defects in DDR. PARP1, ATR–CHK1 signaling, *BRCA1*/*2* and other HR related genes represent key DDR effectors that can be pharmacologically exploited. Despite the significant progress made in this field of DDR therapeutics, major challenges are still open, firstly the acquired resistance observed in these treatments that strongly limits their long-term efficacy [45,46]. In this context, natural products have attracted increasing attention due to their ability to target, directly or indirectly, multiple DDR nodes, including ATR/CHK1 signaling, HR effectors, and cell-cycle checkpoints. Unlike selective synthetic inhibitors, many natural compounds simultaneously induce oxidative stress, replication stress and DNA repair defects, thereby enhancing the vulnerability of breast cancer cells. The following sections discuss the major classes of natural compounds according to their mechanisms of action on the DDR network.

## 4. Natural Compounds as Emerging Resource in Cancer Therapy

The use of natural compounds in oncology treatment stands as a leading translational research field, which holds great promise for the future. During the mid-20th century, scientists discovered vincristine, vinblastine and paclitaxel from plants, which became essential anticancer drugs and proved that natural products could serve as innovative therapeutic agents [47]. Research on marine-derived products, including trabectedin, the microbial compounds anthracyclines and bleomycin, showed that secondary metabolites could develop new therapeutic methods which would expand treatment possibilities [47].

The first description of these compounds dealt with their cytotoxic effects, followed by the emerging studies aimed at testing their potential activity to enhance traditional treatments through chemosensitization and pathway modulation that fights drug resistance [48]. Recently, it has been demonstrated how polyphenols, alkaloids, terpenoids, isothiocyanates and fungal metabolites modulate the key pathways controlling cell survival, cell cycle regulation, and programmed cell death, acting synergistically with chemotherapeutic, targeted agents, and immunotherapy agents [20,48,49]. Emerging evidence shows how the natural compounds reach their best therapeutic results when delivered through nanoparticles, liposomes and hydrogel systems that improve their stability, bioavailability and potency [50]. The new methods transform traditional bioactive metabolites into modern medications functioning as dual-action cancer drugs and treatment response enhancers. Breast cancer treatment now includes naturally occurring molecules as an accepted weapon against the disease; these compounds endowed with therapeutic value derive from different sources including plants, marine and microbial organisms [51]. Here, we review recent advances in the identification of natural products that act along two convergent axes in breast cancer: the direct induction of DNA damage and the modulation of DDR signaling on the one hand, and the modulation of oncogenic signaling and survival pathways on the other. For each chemical class, we discuss the structural features underlying activity, the reported molecular targets, and the evidence supporting the use of these compounds as chemosensitizers or as agents capable of circumventing established resistance mechanisms.

## 5. Polyphenols and Phenolic Derivatives

Polyphenols are a major class of secondary metabolites widely distributed in the plant kingdom [52]. They function as defense elements against both living organisms and environmental stress factors, supporting vital biological functions that include color production, growth management and UV protection. Polyphenols have become a focus of scientific investigation over the last few decades because of their properties as antioxidants, anti-inflammatory, anticancer [53,54] and anti-degenerative in age-related diseases [55,56]. Polyphenols are classified on the basis of their ring structure and functional groups. Accordingly, most dietary polyphenols are grouped in four main subclasses: phenolic acids (hydroxybenzoic acid and hydroxycinnamic acid), flavonoids (flavanols, flavonols, flavones, flavanones, isoflavones, proanthocyanidins and anthocyanins), stilbenes and lignans [57]. Beyond these subclasses, plant phenolics also comprise hydrolysable tannins, such as the ellagitannin castalin, and structurally atypical phenolics, such as the selaginellins; both are considered here in view of their recently reported activity in breast cancer models. Several studies have demonstrated that flavonoids exert anticancer effects at different levels, such as inhibiting cell proliferation, invasion and metastasis [58]. The most well-characterized flavones are Luteolin and Apigenin, which have been extensively studied for their role in breast cancer.

### 5.1. Apigenin (APN)

Apigenin (5,7-dihydroxy-2-(4-hydroxyphenyl) chromen-4-one) is a flavonoid found in several vegetables (onions, celery, spinach, tomatoes, and beans), herbs (peppermint, thyme, basil, oregano, and marjoram) and fruits (grapes, oranges, and apples) [59,60]. Recently, Apigenin has shown a strong therapeutic potential against a certain number of diseases including cardio-vascular, auto-immune, Alzheimer’s, Parkinson’s, and immune-suppressive ones [59]. APN as a phytoestrogen shows estrogen-like activity; it reduces estrogen dominance, inhibiting the proliferation of estrogen-related tumor cells, both in breast and cervical cancers. Moreover, APN impairs cell proliferation of the TNBC cell line, MDA-MB-231, by inducing cell cycle arrest in G2/M phase [61] as a single agent but it also sensitizes both resistant and non-resistant MCF-7 BC cell lines to Doxorubicin (DOX) treatment by downregulating the expression level of MDR1 protein and preventing the phosphorylation of JAK2 and STAT3 [62]. An important role of APN is the induction of DNA damage through the activation of PKCδ, which in turn activates ATM and H2AX, leading to the activation of DDR. In particular, APN does not directly produce ROS or activate caspase 3, but instead induces G1/S phase arrest, thereby increasing apoptotic cells [63]. Despite the use of APN in breast cancer showing promising potential, its clinical application is limited by several aspects such as its poor water solubility, low gastrointestinal absorption and limited systemic bioavailability [60].

### 5.2. Luteolin

Luteolin (2-(3,4-dihydroxyphenyl)-5,7-dihydroxychromen-4-one) is a flavone first isolated from *Reseda luteola* and present in herbs such as honeysuckle, oregano, perilla leaves, peppermint, thyme, rosemary, chrysanthemum, lettuce, and celery. Luteolin has also been identified in lemon, cabbage, fennel, hartwort, beets, spinach, green tea, broccoli, peppers, and dietary sources including wine. Plants rich in luteolin, such as *Terminalia chebula* Retz., have been used in Chinese traditional medicine for treating various diseases such as hypertension, inflammatory disorders, and cancer [64,65,66]. Luteolin has been demonstrated to exhibit antiproliferative effects on both hormone-responsive and TNBC cell lines. However, luteolin was used to reduce the toxicity of DOX in MCF-7 breast cancer cells, but the half-maximal inhibitory concentration (IC_50_) of luteolin is approximately 50 μM, a level that is not physiologically attainable [67]. To address this gap between the concentrations used in pre-clinical studies and those relevant for human application, it is often beneficial to combine two phytochemicals that work synergistically to inhibit cancer cell growth, even when each compound alone does not exhibit antitumor activity at the given dose. A novel combination of luteolin and indole-3-carbinol (I3C) was found to inhibit cell proliferation in estrogen receptor-positive (ER+) breast cancer cell lines MCF7 and T47D in a dose-dependent manner, but not in TNBC cell lines, such as MDA-MB-231 and BT-549. This suggests that the synergistic effect of the two phytochemicals relies on the ERα pathway [68]. The combination of luteolin at 30 μM and I3C at 40 μM (L30I40) effectively inhibited the proliferation of ER+ breast cancer cells, showing similar anticancer effects to commercial drugs like doxorubicin and tamoxifen. Importantly, unlike these drugs, this combination did not affect the viability or morphology of the human endothelial cell line EA.hy926, indicating a potential to reduce side effects. The L30I40 combination inhibits breast cancer cell proliferation by inducing G1 cell cycle arrest through the inactivation of the cyclin D1-CDK4/6 complex; also, it promotes apoptosis by modulating Bcl-xL and Bax protein levels. Luteolin was also explored in a new combination with curcumin, a polyphenolic compound from *Curcuma longa* L. (turmeric), in TNBC cells and cell-derived xenograft mice [69]. The combination of luteolin and curcumin (L30C20) synergistically inhibited cell growth in TNBC cell lines like BT-549 and MDA-MB-231, but not in ER+ breast cancer cells, contrasting with the effects seen when luteolin is combined with I3C. The RNA transcriptome analysis of xenograft tumors indicated that the luteolin-curcumin combination synergistically activated type I interferon (IFN) signaling, while suppressing transforming growth factor-β (TGF-β) signaling. Furthermore, this combination reduced the levels of oncoproteins c-MYC and NOTCH1, which are key regulators of stem cell maintenance, tumor clonal evolution, and drug resistance [69].

### 5.3. Castalin

Castalin (7,8,9,12,13,14,17,18,19,25,29-undecahydroxy-24-(hydroxymethyl)-3,23,26-trioxahexacyclo[13.10.3.1^2,6^.0^5,10^.0^11,28^.0^16,21^]nonacosa-5(10),6,8,11,13,15(28),16,18,20-nonaene-4,22,27-trione) is an ellagitannin, a subclass of hydrolysable tannin found in the shells of *Castanea sativa* and in the leaves of *Melaleuca quinquenervia* which lately has shown to exhibit antitumor activity [70]. Recently, we demonstrated the intriguing role of Castalin in suppressing the growth of breast adenocarcinoma (MCF-7) and triple-negative breast cancer (MDA-MB-231) cells. Castalin induces DNA damage via ROS production [71]. Castalin demonstrates dose- and time-dependent effects on these cancer cell lines, causing DNA damage that activates the mutagenic NHEJ. We also explored how Castalin might enhance the efficacy of the CHK1 inhibitor SRA737 in promoting cancer cell death. The combination of Castalin and SRA737 increases CHK1 Ser345 phosphorylation compared to single treatments, leading to mitotic catastrophe. Through RNA-Seq analysis, the role of Castalin in causing DNA damage was further characterized, confirming its role in HR downregulation and ROS generation [72], by observing the downregulation of both ZNF280A and S1PR1 genes, involved in the DNA end resection process and ROS suppression [73], respectively.

### 5.4. Selaginellin

Selaginellin (4-[[3-(hydroxymethyl)-6-(4-hydroxyphenyl)-2-[2-(4-hydroxyphenyl)ethynyl]phenyl]-(4-hydroxyphenyl)methylidene]cyclohexa-2,5-dien-1-one) is a parent compound that defines a class of structurally unique pigments, selaginellins, that serve as central scaffold for numerous natural products, isolated from the genus Selaginella, particularly *Selaginella tamariscina* (Selaginellaceae) [74]. This plant has a long history in traditional medicine for the treatment of inflammation, dysmenorrhea, chronic hepatitis, and hyperglycemia [74]. The use of these compounds, coupled with the known antitumor effects of *S. tamariscina* in medicine, prompted the investigation of its use against breast cancer [75]. A recent work by Wen et al. studied the chemical diversity of selaginellin from *S. tamariscina* to discover potential anti-breast cancer agents [76]. In particular, the constituents of the 75% ethanol extract of the aerial parts of this species were examined, and seventeen selaginellin derivatives, including five previously undescribed compounds, were isolated and tested against breast cancer. This study revealed that dimeric selaginellin derivatives, especially diselaginellins B, showed stronger antitumor activity than 5-FU in TNBC cell lines, such as MDA-MB-468 and MDA-MB-231 cells, leading to an increased phosphorylation of Chk1 and Cdc25c, favoring the G2/M phase arrest. Moreover, treatment of MDA-MB-231 cells with diselaginellins B caused an induction of apoptosis and an increase in ROS generation in a concentration-dependent manner [76].

**Figure 3:**
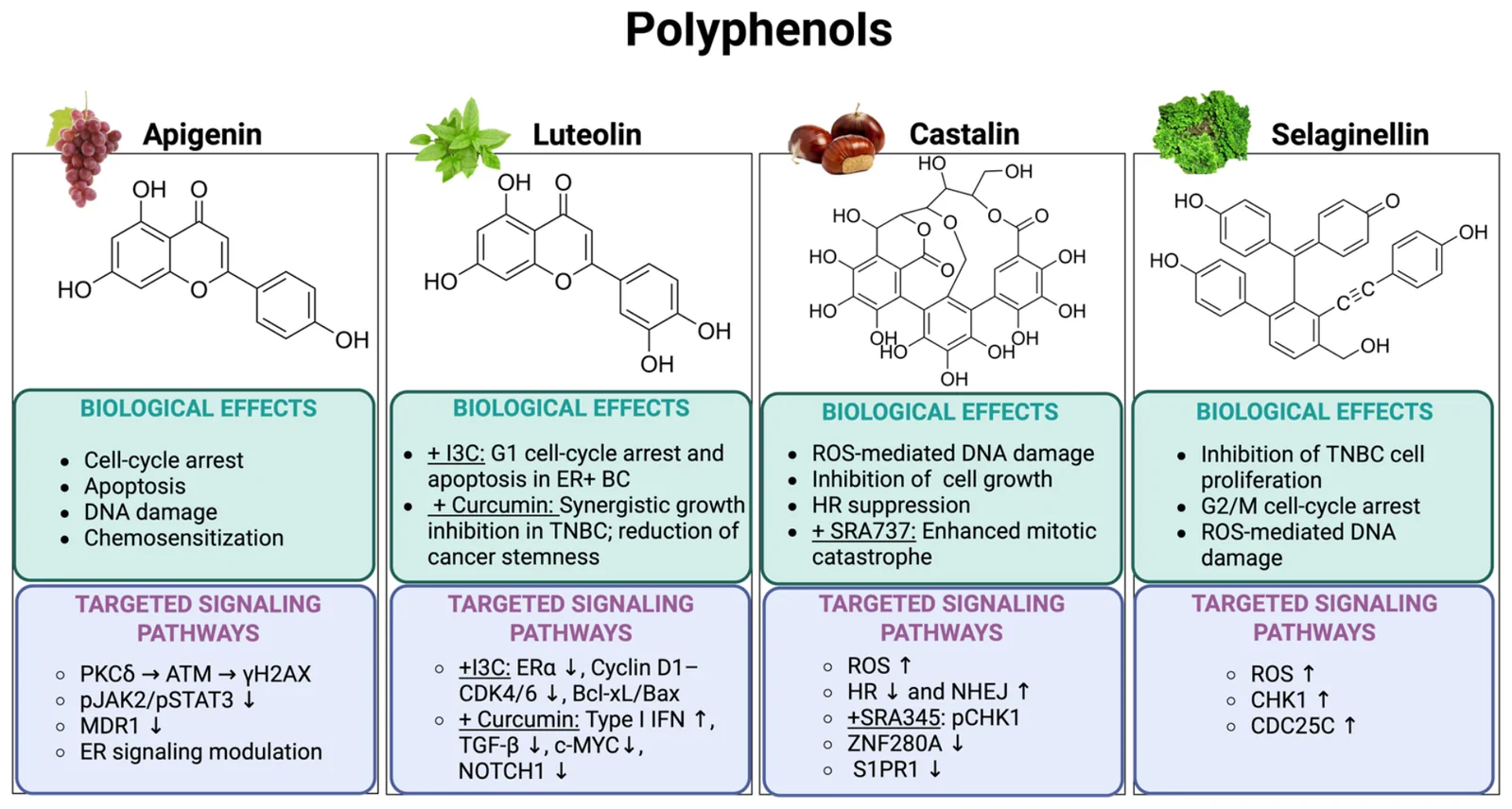
Natural polyphenols and their role in breast cancer therapy. The figure summarizes the selected polyphenols discussed above, with reported activity against breast cancer highlighting their chemical structures, natural sources, and principal anticancer mechanisms, including modulation of DDR signaling, induction of cell-cycle arrest, generation of reactive oxygen species (ROS), inhibition of homologous recombination, and enhancement of sensitivity to targeted therapies. Created in BioRender. De Laurentiis, M. (2026) https://BioRender.com/11x8ncw.

Polyphenols emerge as a multifunctional class of compounds that, despite their structural diversity, share the ability to interfere with cell-cycle progression and modulate DDR (Figure 3). APN primarily activates the ATM-mediated DNA damage response and induces cell cycle arrest in Breast cancer cells. Both castalin and selaginellin promote ROS-dependent DNA damage, triggering replication stress and activation of the DDR. In addition, castalin selectively reshapes DNA repair pathway choice by downregulating HR and favoring the error-prone NHEJ pathway, thereby exacerbating genomic instability and DNA damage accumulation. In contrast to apigenin, castalin and selaginellins, whose antitumor activity is closely linked to the induction of DNA damage and DDR activation, luteolin exerts its therapeutic effects mainly through the modulation of key oncogenic signaling pathways. Specifically, in combination with indole-3-carbinol (I3C), luteolin suppresses ERα signaling and inhibits the proliferation of ER-positive breast cancer cells. In contrast, its combination with curcumin preferentially targets TNBC by activating IFN signaling while inhibiting TGF-β signaling, an immunomodulatory strategy already recognized as a promising therapeutic approach in solid tumors [77]. Together these findings support the potential of polyphenols as therapeutic agents in breast cancer therapy both as single agents and in adjuvant therapies. Both apigenin and luteolin enhance the therapeutic efficacy of Doxorubicin, supporting their potential as chemosensitizer agents in current breast cancer therapies.

## 6. Terpenoids and Derivatives

Terpenoids constitute the largest and most diverse class of natural compounds, with more than 50,000 known molecules identified extensively across both the plant and fungal kingdoms [78]. The chemical structure of these compounds is formally defined by the number of isoprene units contained in the single molecule [78]. Terpenes originate from the mevalonic acid and methyl erythritol 4-phosphate biosynthetic pathways and are classified based on carbon skeleton size, primarily as hemiterpenes with a single isoprene group (C_5_), monoterpenes (C_10_) when two isoprene units are linked, sesquiterpenes (C_15_), diterpenes (C_20_), sesterterpenes (C_25_), triterpenes (C_30_), and tetraterpenes (C_40_) [78]. Terpenes play fundamental roles in plants, where they act as defensive elements against pests and environmental stress, and are the principal constituents of essential oils [79]. Due to their extensive and potent biological profiles, terpenes and their derivatives are extensively used in pharmaceutical and industrial applications, ranging from fragrances and flavors to active ingredients in modern medicines. Over the past few decades, terpenes have become a major focus of scientific investigation due to their documented pharmacological activities, which include antioxidant, antimicrobial, antiviral, anti-inflammatory, antiparasitic, antidiabetic, and significant antitumor effects [80]. The following section examines individual terpenoid compounds and their derivatives that scientists have studied recently in breast cancer models to determine their molecular targets and biological effects.

### 6.1. Oleanolic Acid (OA)

Oleanolic acid (4aS,6aR,6aS,6bR,8aR,10S,12aR,14bS)-10-hydroxy-2,2,6a,6b,9,9,12a-heptamethyl-1,3,4,5,6,6a,7,8,8a,10,11,12,13,14b-tetradecahydropicene-4a-carboxylic acid, OA) is a pentacyclic triterpenoid widely distributed across over 1600 plant species. Its name originates from the Oleaceae family, as it is notably abundant in the leaves and fruits of the European olive (*Olea europaea*), as well as in grapes (*Vitis vinifera* L.), bilberries, and apples [81]. In plants, this compound exerts essential functions, such as preventing water loss and acting as a primary defense against pathogens and herbivores. Historically, this compound has been extensively used in traditional Chinese medicine for its hepatoprotective properties, and it remains an established over-the-counter liver protective agent in Asia. OA is renowned for its broad spectrum of pharmacological activities, including potent antioxidant, antimicrobial, antiviral, anti-inflammatory, and antidiabetic effects as well as the improvement of mitochondrial activity in C2C12 myoblast cells [81]. Furthermore, OA has been previously recognized as a metabolite involved in modulating resistance to radiotherapy in TNBC models, consolidating its role as a potential chemosensitizer [82]. In particular, the combination of OA with radiation (4 Gy) and Olaparib significantly enhances the sensitivity of the TNBC cell line, MDA-MB-231, to radiation, thereby inhibiting cell proliferation [82]. Consistently, in one of our recent studies, we have demonstrated that OA increased the activity of Camptothecin (CPT), a chemotherapeutic inhibitor of Topoisomerase I, thus increasing cancer cell death [83]. Specifically, OA was shown to alter the DNA repair pathway choice in combination with CPT by reducing HR activity and promoting a more rapid but mutagenic single-strand annealing (SSA) pathway. These findings suggest that OA increases the efficacy of CPT by directing DNA repair through an error-prone process, thereby enhancing cancer cell genomic instability, which results in cell death. This study emphasizes the potential of OA as an adjuvant compound, particularly given its non-cytotoxic nature at the low doses examined [83].

### 6.2. Ent-Abietane Diterpenoids

*Ent*-Abietane diterpenoids are the main bioactive constituents identified and extracted from the dried root of the Euphorbiaceae family, such as *Euphorbia fischeriana* Steud., a perennial herbaceous plant used for millennia in traditional Chinese medicine for the treatment of lymphoid tuberculosis, psoriasis and ringworm [84,85]. These compounds belong to the class of natural diterpenoids, characterized by a chemical structure based on the *ent*-abietane scaffold, which show cytotoxicity against human prostate cancer [85]. Furthermore, phytochemical investigation of *E. fischeriana* has led to the isolation of a significant number of these molecules with anticancer activity against breast cancer [86]. As a broader family of compounds, they have been described as inducers of DNA damage in cancer cells. In particular, *ent*-abietane diterpenes, such as 19-(Benzyloxy)-19-oxojolkinolide B (19-BJB), have been shown to interact directly with DNA, leading to the activation of checkpoint kinase 1 (CHK1) and checkpoint kinase 2 (CHK2) in bladder cancer cell lines [87]. Recently, two other molecules from the *Ent*-Abietane Diterpenoids family, 17-hydroxyljolkinolide B and 17-acetyljolkinolide B, were described to exhibit the most potent cytotoxic effects across a panel of human breast cancer cell lines, including MCF-7, ZR-75-1 and MDA-MB-231, with a particular enhanced activity against the highly aggressive TNBC cell line, MDA-MB-231 [86]. The diterpenoids differ mainly in specific functional groups, allowing the authors for the evaluation of their contribution to cytotoxicity. For instance, comparisons between jolkinolide A and jolkinolide B, as well as between their respective 17-hydroxylated derivatives, revealed that the presence of an epoxy group at the C11–C12 position plays a crucial role in enhancing their biological activity.

**Figure 4:**
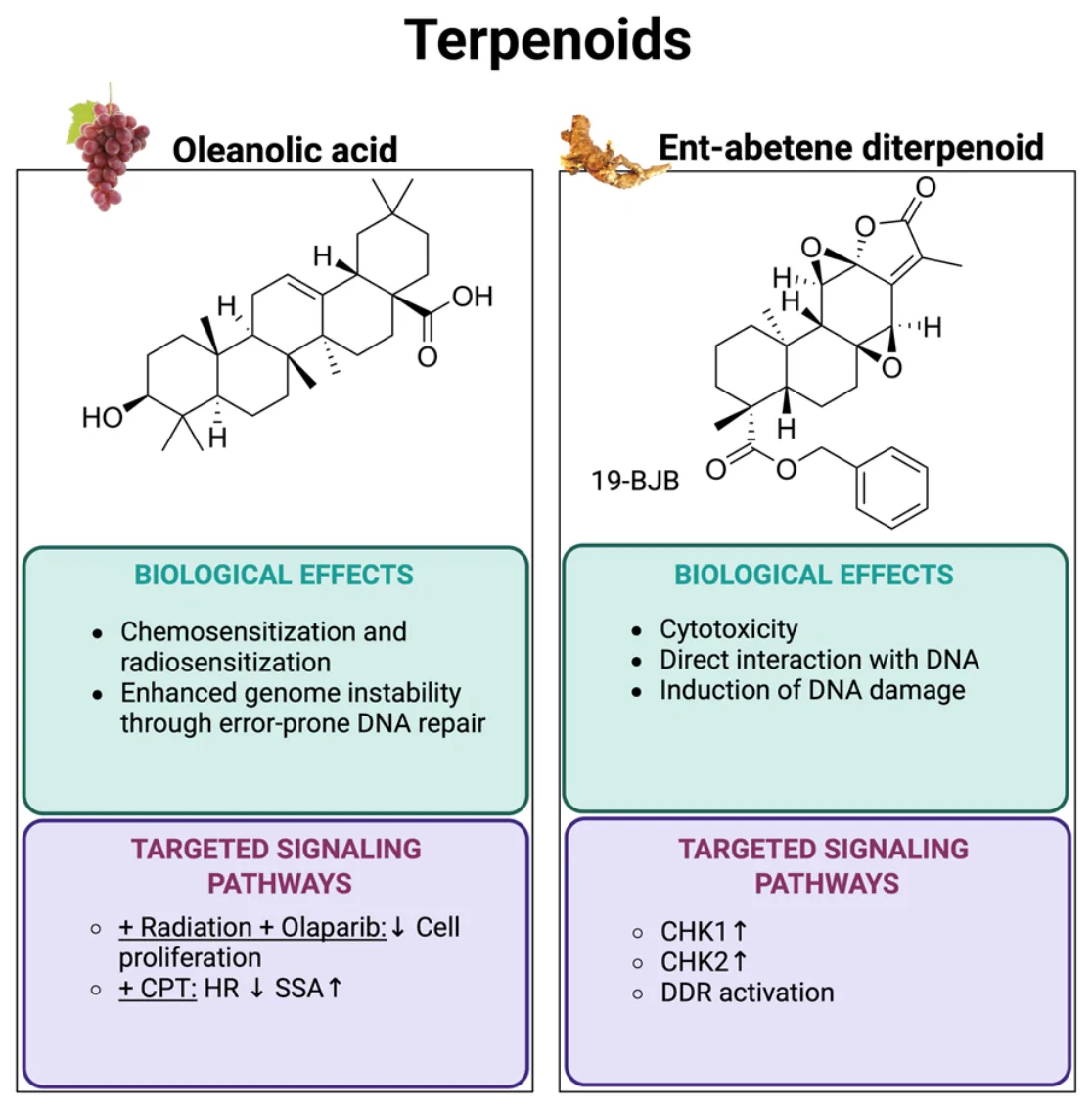
Oleanolic acid and *ent*-abietane diterpenoid (19-BJB) as natural modulators of DNA damage response signaling in breast cancer. The figure summarizes the chemical structures, natural sources, and major anticancer activities of oleanolic acid and *ent*-abietane diterpenoid (19-BJB) in breast cancer. Oleanolic acid exerts its antitumor effects through modulation of DNA damage response (DDR) pathways, induction of cell-cycle arrest, and enhancement of chemosensitivity, while 19-BJB exerts its effects through direct interaction with DNA and promotion of DNA damage. Created in BioRender. De Laurentiis, M. (2026) https://BioRender.com/fleia6e.

In summary, the activity of these two terpenoid compounds led to breast cancer cell death with different mechanisms of action (Figure 4). Oleanolic acid acts predominantly as a chemosensitizer by modulating DDR in combination with DNA-damaging agents, such as radiotherapy and camptothecin. Specifically, it promotes more error-prone pathways, enhancing genomic instability and therapeutic efficacy. In contrast, ent-abietane diterpenoids primarily exert their antitumor activity by inducing DNA damage through direct interaction with DNA.

## 7. Alkaloids

Alkaloids are one of the most prominent classes of secondary metabolites primarily produced by plants. They are extremely abundant in flowering plants (Angiospermae) as salts of organic acids (acetic, lactic, malic, tartaric, oxalic, aconitic, and tannic acids) in different parts of plants such as leaves, bark, seeds and roots [88]. Besides plants, alkaloids are also found in other biological kingdoms, including fungi such as psilocybin in the *Psilocybe* genus and in some animals, like bufotenin found in the skin of certain toads and insects. Furthermore, many marine organisms such as cyanobacteria are also known to contain alkaloids [89]. Chemically, alkaloids can be defined as a large and diverse group of nitrogen-containing specialized molecules characterized by the presence of at least one nitrogen atom within a heterocyclic ring [90]. Their chemical structure is responsible for their distinctive chemical reactivity and potent biological properties. Due to their diverse and potent biological profile, alkaloids are highly valued for pharmaceutical applications as they possess a wide range of properties including anti-inflammatory, antimicrobial and antiprotozoal effects [90]. As an example of their therapeutic potential, quinine, a cinchona alkaloid first isolated in 1820 from a native cinchona tree in Peru, was the major antiprotozoal used in the treatment of malaria. Moreover, as a testament to their diverse bioactivities, a recent study has revealed that alkaloids like talimonine (from *Thalictrum simplex*) and sofalin D (from *Sophora alopecuroides*) can prevent the viral replication of SARS-CoV-2 [91]. Similarly, cryptosminrin, cryptospirolepine, and biscriptolepine (from *Cryptolepis sanguinolenta*) were also identified as potential inhibitors of SARS-CoV-2 viral proteins [92]. The alkaloids are quite toxic to humans [93]; for example, psilocin possesses psychotropic activity, while compounds such as cocaine, caffeine, nicotine, and theobromine exhibit stimulant effects. The following section examines individual alkaloids, which scientists have recently studied in breast cancer models to determine their molecular targets and biological effects.

### 7.1. Cyclovirobuxine D

Cyclovirobuxine D (1S,3R,6S,8R,11S,12S,14R,15S,16R)-15-[(1S)-1-(dimethylamino)ethyl]-7,7,12,16-tetramethyl-6-(methylamino)pentacyclo[9.7.0.01,3.03,8.012,16]octadecan-14-ol (CVB-D) is a natural alkaloid extracted from the herb *Buxus sinica* [94], showing anticancer activity [95]. More recently, CVB-D has been demonstrated to reduce cell proliferation by strongly downregulating the expression of the nuclear antigen Ki-67 in TNBC cells (MDA-MB-231, BT-549, Hs578T), compared with human normal breast epithelial MCF-10A cells [96]. Consistently, CVB-D directly binds to YAP, leading to decreased YAP/TAZ nuclear translocation and increased p-YAP levels in TNBC. Phosphorylation of YAP prevents its nuclear translocation, essential for its oncogenic functions, impairing the activation of its downstream oncogenic target genes such as *CTGF*, *CYR61*, and *c-Myc* [96]. The antitumor efficacy of CVB-D against TNBC was also evaluated in vivo using an MDA-MB-231 xenograft mouse model; treatment with CVB-D (5 or 10 mg/kg) significantly reduced tumor volume and weight, showing a stronger effect than the positive control verteporfin (VP), an FDA-approved anticancer agent that serves as a potent YAP inhibitor [96]. In regard to DNA damage, CVB-D is involved in several important processes [97]. First, CVB-D has been shown to reduce Doxorubicin (DOX)-induced cardiotoxicity. Pre-treatment with CVB-D attenuated DOX-induced cardiac contractile dysfunction and modulated the ratio of reduced glutathione (GSH) to oxidized glutathione (GSSG) [97]. Moreover, CVB-D has been reported to induce apoptosis through DNA damage and impairment of DNA repair mechanisms in castration-resistant prostate cancer, one of the deadliest malignancies with limited therapeutic options. Specifically, CVB-D reduced cell viability in PC3 and C4-2 cell lines. RNA-seq analysis revealed that CVB-D modulates several biological pathways, among which mismatch repair complex binding, mismatched DNA binding, and damaged DNA binding were the most significantly enriched processes. Using the MCC algorithm in cytoHubba, the authors identified key hub nodes from the RNA-seq data, revealing a core target network characterized by BRCA1, POLD1, BLM, MSH2, MSH6, and PCNA [98].

### 7.2. Piperine

Piperine ((2*E*,4*E*)-5-(1,3-benzodioxol-5-yl)-1-piperidin-1-ylpenta-2,4-dien-1-one, PIP) is the main natural amide alkaloid isolated from the fruits of plants belonging to the Piperaceae family, primarily *Piper nigrum*, commonly known as black pepper [99]. Beyond its primary role as the source of black pepper’s flavor, this compound has garnered substantial attention from medicinal chemists and health professionals due to its extensive array of therapeutic benefits, including antioxidant, antitumor, anti-inflammatory, anti-asthmatic, hepatoprotective, immunomodulatory, and antibacterial properties [99]. In the context of cancer therapy, piperine has exhibited inherent anticancer activity by targeting several molecular pathways [100], particularly in combination with the conventional chemotherapeutic drug Doxorubicin, demonstrating a potentiated therapeutic effect in both in vitro and in vivo studies [99]. Hakeem et al. demonstrated that PIP increased the sensitivity of the TNBC cell line MDA-MB-231 to DOX treatment [99]. Mechanistically, PIP treatment efficiently reduced the expression of aldehyde dehydrogenase 1 (ALDH1), a functional stem cell marker commonly used to identify the cancer stem cell (CSC) population in different tumors [101]. Notably, the combined treatment with DOX resulted in a stronger reduction in this population in MDA-MB-231 cells compared to single PIP treatment. This effect was further validated by immunohistochemistry analysis of tumor tissue sections derived from an Ehrlich ascites carcinoma (EAC) solid tumor animal model. The combination of PIP and DOX also leads to a strong reduction in the level of p-AKT and mTOR compared to either treatment alone, both in vitro and in vivo, suggesting inhibition of the PI3K/AKT/mTOR pathway. Moreover, it was assessed that PIP exerts a protective effect against DOX-induced cardiotoxicity, since the co-treatment group showed more intact cardiac muscle cells with a smaller number of degenerated cells harboring pyknotic nuclei. These findings are consistent with previous studies reporting that PIP alleviates DOX-induced cardiotoxicity in mice by activating the peroxisome proliferator-activated receptor γ (PPAR-γ) [102].

**Figure 5:**
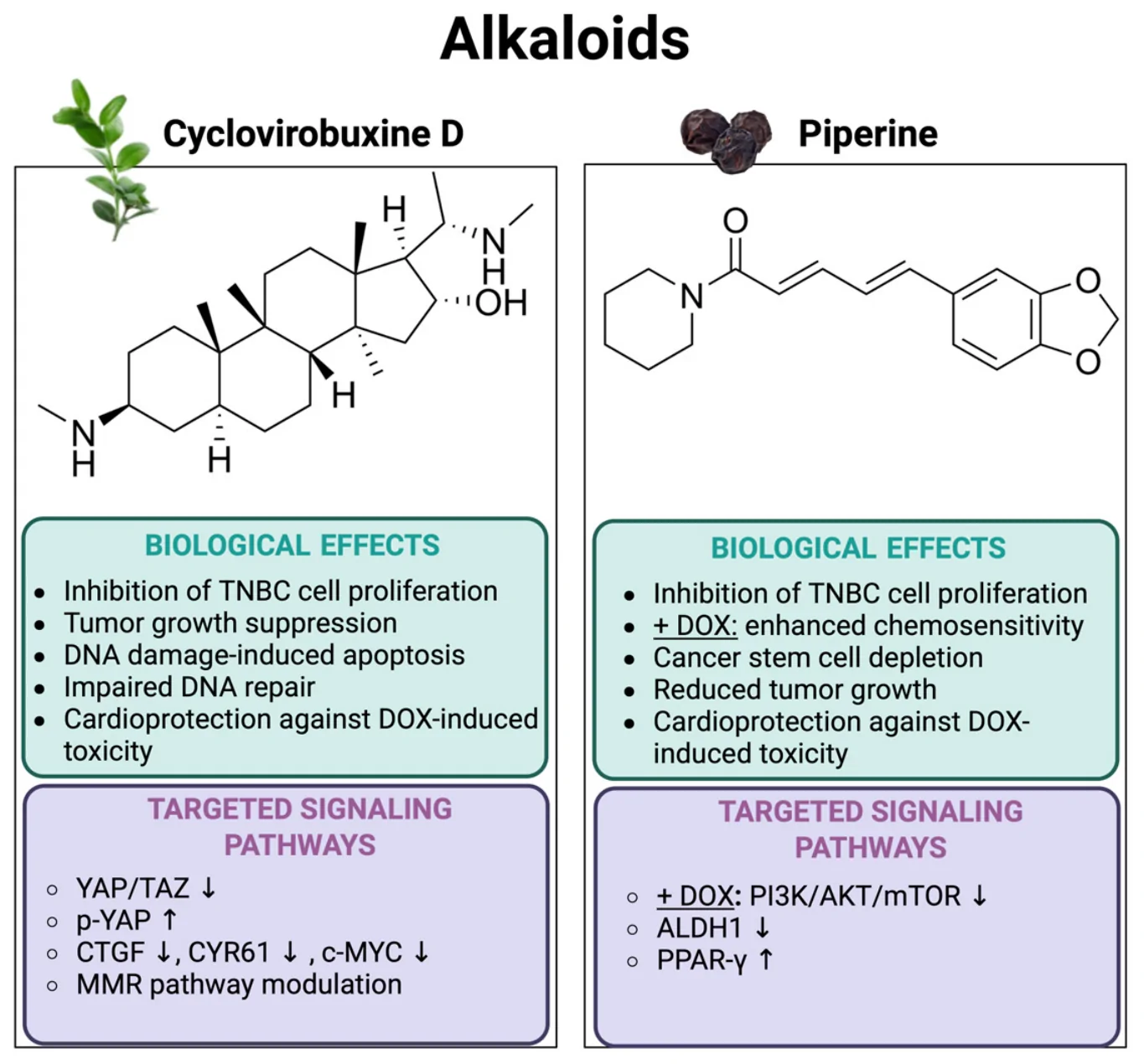
Natural alkaloids with reported antitumor activity against breast cancer. The figure summarizes selected alkaloids with reported antitumor activity in breast cancer, highlighting their chemical structures, natural sources, and principal mechanisms of action. Both compounds show chemosensitizing activity and the potential to mitigate doxorubicin-associated cardiotoxicity, supporting their use as promising adjuvants in combination therapeutic strategies. Cyclovirobuxine-D also exerts its anticancer effects through the induction of DNA damage and inhibition of cell proliferation. Created in BioRender. De Laurentiis, M. (2026) https://BioRender.com/wy2yc73.

The alkaloids discussed in this section exert their anticancer activity through multiple mechanisms (Figure 5); cyclovirobuxine D interferes with the YAP/TAZ oncogenic signaling pathway by directly inhibiting YAP nuclear translocation and so suppressing its oncogenic function. Moreover, in a prostate cancer model, CVB-D has been shown to affect genome integrity by impairing DNA damage repair, but the anti-breast cancer activity remains to be determined. Piperine primarily acts as a chemosensitizer by targeting cancer stem cell populations and inhibiting pro-survival signaling pathways, such as the PI3K/AKT/mTOR pathway, enhancing the efficacy of conventional chemotherapy. Notably, both compounds show protective effects against doxorubicin-induced cardiotoxicity, highlighting their potential as promising adjuvant agents for BC therapy.

## 8. Sulfur-Containing Compounds

Sulfur-containing compounds are a prominent class of secondary metabolites widely distributed throughout the plant kingdom, characterized by the presence of a sulfur atom in various oxidation states [103]. These molecules constitute a broad and chemically diverse group of metabolites [104], performing essential functions for plant life as defense elements against pathogens and environmental stress [105]. Among them, two major subclasses of plant-derived sulfur-containing compounds are Organosulfur Compounds (typical of *Allium* species) and Glucosinolates (characteristic of Brassicaceae), which differ in both their chemical structures and biosynthetic origin [104]. The main dietary sources of these compounds are plants belonging to the Alliaceae family (such as garlic, onion, and chives) and the Brassicaceae family (such as broccoli, cabbage, and horseradish) [106]. Sulfur-containing compounds are highly valued in the pharmaceutical and nutraceutical fields for their powerful and broad biological activities, which include anticancer, anti-aging, antimicrobial, antioxidant, and anti-inflammatory ones [107]. As proof of their therapeutic potential, sulforaphane (which is derived from glucosinolates) is extensively studied for its chemopreventive properties by inducing detoxification enzymes [108], while allicin (an organosulfur compound) is known as a browning agent for adipocytes [109]. The following section examines sulfur-containing compounds which scientists have recently studied in breast cancer models to determine their molecular targets and biological effects.

### 8.1. Erucin

Erucin (1-isothiocyanato-4-methylsulfanylbutane) is an isothiocyanate glucosinolate hydrolysis product structurally related to sulforaphane. It is a bioactive metabolite derived from cruciferous vegetables, particularly rocket salad (*Eruca sativa*), and it is known for its anti-inflammatory, antihypertensive, chemopreventive, vasorelaxant, and anticancer properties [110]. The anticancer potential of Erucin has been extensively investigated in several tumor models, demonstrating its ability to inhibit cell proliferation and modulate cell cycle progression to induce apoptosis in prostate, lung, liver and colon cancer cells [111,112]. In regard to breast cancer, the effect of Erucin was evaluated across multiple cell lines representing distinct molecular subtypes of breast cancer, including T47D (ER-positive cell line) and MDA-MB-231 (TNBC cell line) [113,114,115]. Erucin effectively impairs the cell viability of all the breast cancer cells, although the ER-positive T47D cell line exhibited lower sensitivity. Importantly, Erucin showed a weaker effect on normal epithelial breast cells, MCF10A, suggesting a degree of selectivity for malignant cells [116]. Given the growing interest in overcoming chemoresistance, Erucin has been explored in nano formulation-based combinational drug delivery systems. In particular, co-encapsulation of Erucin in combination with paclitaxel into a nanoemulsion formulation with frankincense oil has demonstrated promising therapeutic potential [117]. The optimized nanoemulsion of a paclitaxel and erucin combination (EPNE) not only provided sustained release but also exhibited improved efficacy in inhibiting the growth of paclitaxel-resistant estrogen-positive human breast cancer cells, specifically the T47D cell line. Furthermore, the EPNE treatment resulted in a stronger reduction in tumor size compared to PTX or ER alone in the breast cancer model induced by 7,12-Dimethylbenz(α)anthracene (DMBA) in the Balb/c mice [117]. In a more recent study, Erucin’s mechanism of action has been well characterized against triple-negative breast cancer cells [118]. Erucin has been shown to induce both apoptosis and autophagy in MDA-MB-231 cell lines, as evidenced by the cleavage of Caspase-3 and PARP1, along with the increased expression of key autophagy regulatory genes, such as ULK1, ATG13, BECN1, and BNIP3 [118]. Additionally, Erucin enhances the expression of LC3II, a key positive regulator of autophagosome formation, while it reduces the expression of the negative regulator p62. Erucin treatment significantly inhibited the intracellular formation of ROS in TNBC cells [118]. This effect might be attributed to the capability of the hydrogen sulfide (H_2_S) released intracellularly by Erucin to directly scavenge ROS [119]. Furthermore, treatment with 30 μM Erucin markedly increased the expression of genes encoding antioxidant enzymes, including heme oxygenase-1 (HMOX-1), Glutamate–Cysteine Ligase Catalytic Subunit (GCLC), Glutamate–Cysteine Ligase Modifier Subunit (GCLM), and Superoxide Dismutase 1 (SOD1).

### 8.2. Diallyl Trisulfide

Diallyl Trisulfide (3-(prop-2-enyltrisulfanyl)prop-1-ene, DATS) is an organosulfur compound derived from garlic (*Allium sativum*), which is a widely studied plant belonging to the Alliaceae family [120]. This compound is well-known for its selection of biological properties such as anticancer and chemopreventive against a variety of malignancies [121]. A study conducted by Chang et al. demonstrated that diallyl trisulfide significantly enhanced the cytotoxicity of Doxorubicin in breast cancer cells, revealing a specific mechanism of action [122]. One of the primary mechanisms contributing to chemoresistance in breast cancer is metabolic reprogramming, such as the well-known Warburg effect. In this process, cancer cells favor aerobic glycolysis over oxidative phosphorylation to support their rapid proliferation and survival [123]. A recent study has indicated that combining DATS with low doses of DOX not only enhances DOX’s chemosensitivity—markedly decreasing the viability of both MCF7 and MDA-MB-231 cells—but also significantly reduces glucose uptake in these cell lines [122]. Notably, this combination treatment effectively suppresses essential glycolytic regulators such as glucose transporter 1 (GLUT1), lactate dehydrogenase A (LDHA), and hypoxia-inducible factor-1 alpha (HIF-1α), while concurrently increasing the expression of pyruvate dehydrogenase (PDH) in both MCF7 and MDA-MB-231 cells. To assess the therapeutic efficacy of the DATS and DOX combination treatment in vivo, luciferase-expressing MDA-MB-231 breast cancer cells were implanted into the fourth mammary fat pad of NOD/SCID mice. The combined treatment led to a significant reduction in tumor volume compared to single-drug treatments and the control group. Moreover, mice receiving the combination treatment showed significantly prolonged survival in comparison to other groups.

**Figure 6:**
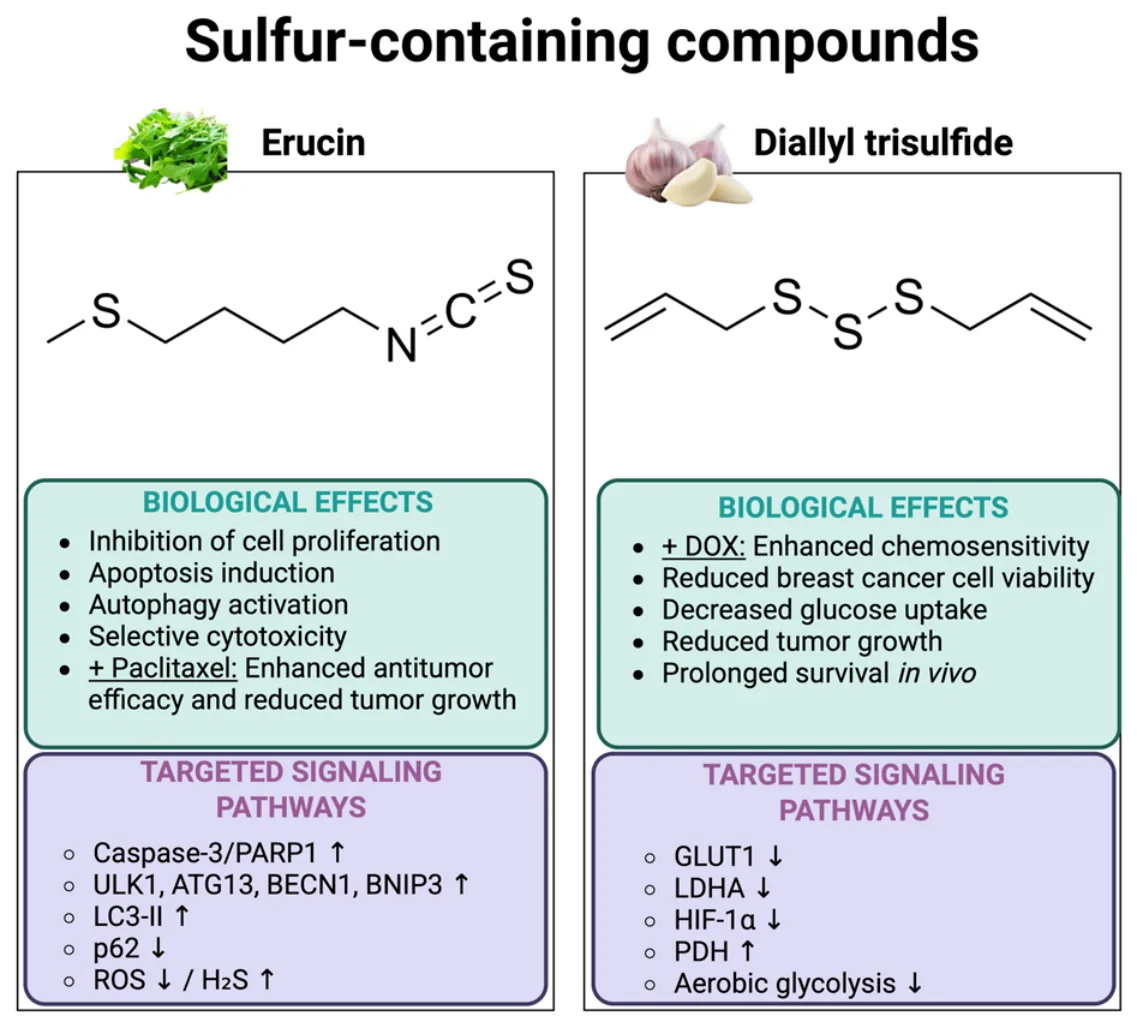
Sulfur-containing compounds and their anticancer activities in breast cancer. The figure summarizes the chemical structure, natural sources and principal anticancer activities of Erucin and Diallyl trisulfide in breast cancer. Erucin exerts antitumor effects through the induction of apoptosis and autophagy and has demonstrated promising efficacy in combination with Paclitaxel. Diallyl trisulfide displays chemosensitizing properties when combined with Doxorubicin (DOXO), and it is also able to modulate tumor metabolism by reducing glucose uptake. Created in BioRender. De Laurentiis, M. (2026) https://BioRender.com/1hhav04.

Sulfur-containing compounds exert their anti-breast cancer activity mainly by enhancing the efficacy of conventional therapies (Figure 6). Erucin promotes apoptosis and autophagy, potentiating the antitumor effects of paclitaxel while reducing intracellular ROS levels through its antioxidant activity. In contrast, Diallyl Trisulfide acts as a chemosensitizer to doxorubicin by overcoming chemoresistance through metabolic reprogramming, suppressing glucose uptake and aerobic glycolysis. Consistently, their ability to sensitize breast cancer cells to standard treatments highlights their potential as promising adjuvant agents in combination therapies.

## 9. Other Emerging Natural Compounds and Their Bioactive Updates

In addition to the major classes of secondary metabolites (polyphenols, terpenes, alkaloids, and sulfur-containing compounds), scientific investigation in cancer research has recently focused on compounds with unique molecular structures or on naturally derived nanoplatforms [124]. A new area of research is exploiting the ability of plants to produce Exosome-Like Nanovesicles; plant-derived extracellular vesicles represent a promising biotherapeutic agent in cancer due to their negligible systemic toxicity, high bioavailability, and cost-effectiveness compared to artificial nanovesicles. These natural nanoparticles, which typically have a diameter below 200 nm, can deliver incorporated lipids, proteins, and nucleic acids, including microRNA, to mammalian cells. They thus represent a significant potential nanoplatform for the transfer of biological molecules that induce anticancer activity, and, for this reason, recent studies have highlighted the potential of these nanovesicles in the therapy of triple-negative breast cancer and other aggressive tumors.

### 9.1. Plant-Derived Extracellular Vesicles (PEVs) from P. grandiflorum (PGEVs)

*Platycodon grandiflorum* (Jacq.) A.DC. is a perennial herb belonging to the Campanulaceae family, widely studied for its anti-inflammatory, antioxidant, hepatoprotective, anticancer, and immunomodulatory properties [125]. Platycodin D (PD), the primary active component of PG, has demonstrated broad-spectrum antitumor activity across multiple tumors, including glioma, oral cancer, breast cancer, gastric cancer, non-small cell lung cancer, liver cancer, leukemia, melanoma and bladder cancer [126,127]. However, the clinical application of Platycodin D is limited by its poor solubility and low oral bioavailability; to address these limitations, a recent study has explored plant-derived extracellular vesicles (PEVs) from *P. grandiflorum* (PGEVs) as an anticancer compound [128]. PGEVs displayed significant anticancer activity in vitro, particularly against the mouse TNBC cell line 4T1, by inhibiting tumor cell proliferation and inducing tumor cell apoptosis [128]. Treatment with PGEVs led to a substantial increase in intracellular ROS levels, suggesting oxidative-stress mediated cytotoxicity. In the TNBC mouse model, PGEVs administered both orally and intravenously resulted in significant inhibition of tumor growth, increased apoptosis and reduced angiogenesis, as indicated by the decrease in the angiogenesis marker CD31 expression [128]. Moreover, PGEV treatments modulated the TME by decreasing PD-1 expression in tumors; boosting the antitumor activity of CTLs; and increasing the concentrations of pro-inflammatory cytokines TNF-α, IL-6 and IFN-γ in the serum. These findings suggest that PGEVs could effectively activate and regulate the immune response and enhance the immunotherapeutic effect of TNBC.

### 9.2. Bitter Melon-Derived Vesicle-like Nanostructures (BMVEs)

Bitter melon is a medicinal and edible plant used as a folk remedy with antitumor and anti-inflammatory effects [129]. Both in vitro and in vivo studies have shown that bitter melon extract (BME) exerts antitumor effects on oral squamous cell carcinoma (OSCC) [130] and head and neck squamous cell carcinoma (HNSCC) [131]. More recently, attention has shifted toward bitter melon-derived vesicle-like nanostructures as potential therapeutic agents. A 2023 study demonstrated that BMVEs exhibit significant antiproliferative and anti-migratory effects in breast cancer models [132]. BMVEs uptake by tumor cells was shown to be both time- and dose-dependent, as evidenced in murine 4T1 cells, with increased internalization correlating with enhanced antitumor activity. Treatment with BMVEs significantly inhibited the proliferation of both murine 4T1 and human MCF7 breast cancer cells and reduced migratory capacity, particularly in 4T1 cells. The anticancer effects of BMVEs appear to be associated with their induction of ROS, leading to apoptosis in a dose- and time-dependent manner. Notably, ROS generation was significantly lower in non-tumorigenic breast epithelial cells (MCF10A), suggesting selective cytotoxicity toward cancer cells [132]. These effects were also confirmed in 3D-cultured 4T1 cells, supporting the translational relevance of BMVEs. The safety of BMVEs was tested in vivo in a 4T1 tumor-bearing mouse model; everyday intratumorally administration of BMVEs caused a significant reduction in tumor volume and weight compared to controls [132]. This antitumor activity was accompanied by increased DNA damage, likely attributable to ROS-mediated mechanisms. Importantly, no significant systemic toxicity was observed. Collectively, these findings point to BMVEs as a promising natural nanomedicine with potent anticancer activity against breast cancer. Such plant-derived vesicle systems may represent an emerging class of biocompatible and effective platforms for cancer therapy, warranting further investigation for clinical applications.

**Figure 7:**
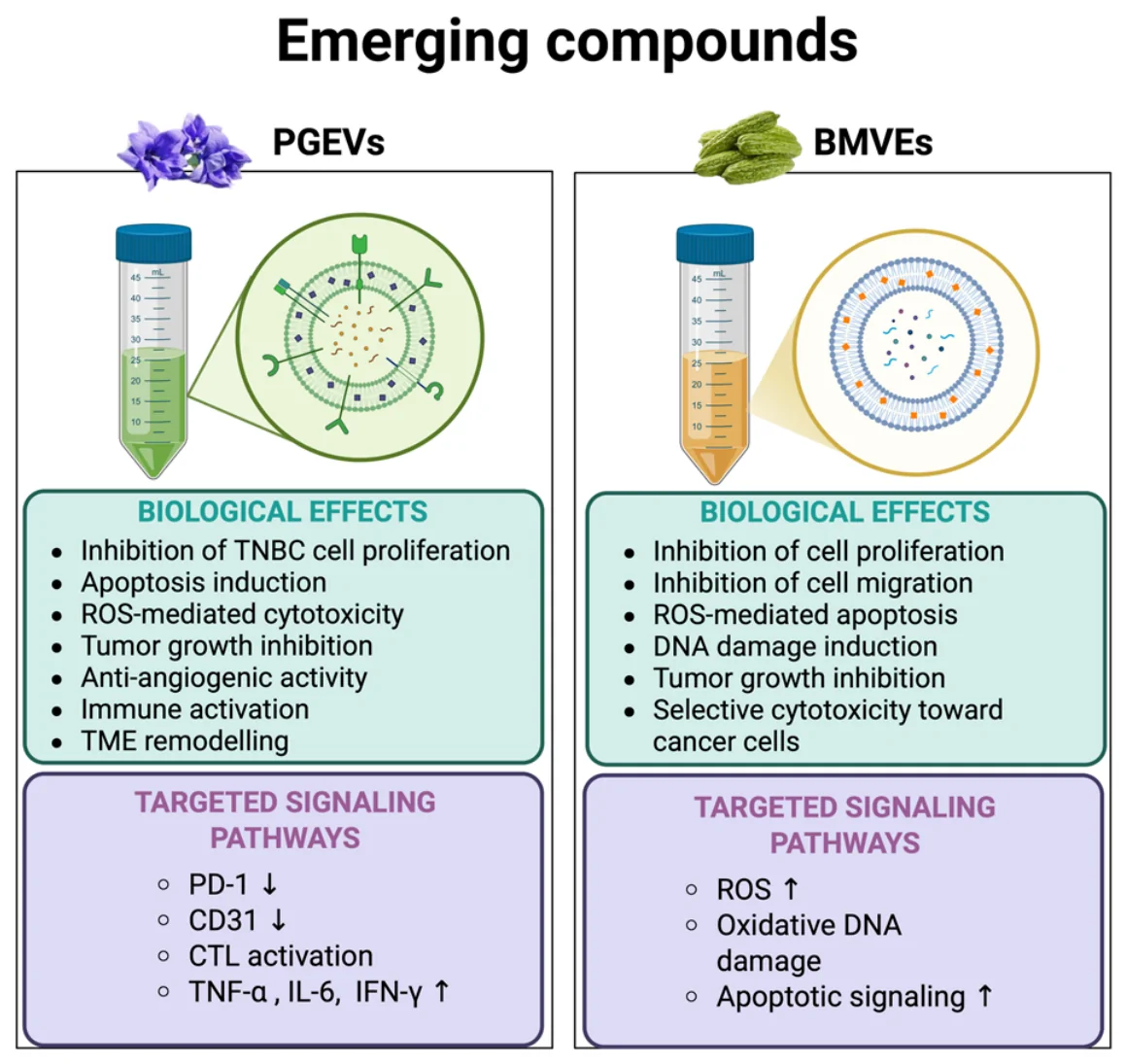
Emerging compounds: plant-derived extracellular vesicles (PEVs) from *P. grandiflorum* (PGEVs) and bitter melon-derived vesicle-like nanostructures (BMVEs). The figure summarizes the main anticancer activities of PGEVs and BMVEs in breast cancer. PGEVs exert antitumor effects by inducing apoptosis and DNA damage through ROS production. In addition, they show promising activity in modulating the tumor microenvironment (TME), including deregulation of PD-1 expression in tumor cells. BMVEs also induce DNA damage via ROS generation and display anti-migratory properties. Notably, this compound has demonstrated a favorable safety profile in in vivo studies. The plant-derived vesicle systems may represent an emerging class of biocompatible and effective platforms for cancer therapy. Created in BioRender. De Laurentiis, M. (2026) https://BioRender.com/9svuksi.

Plant-derived extracellular vesicles represent an emerging class of natural nanotherapeutics with potent anticancer activity and minimal systemic toxicity. Both PGEVs and BMVEs induce ROS-mediated DNA damage, thereby promoting oxidative stress and tumor cell death (Figure 7). Notably, PGEVs directly modulate the TME, a key determinant of therapeutic response, by enhancing antitumor immune activity. This dual mechanism highlights its potential both as a direct anticancer agent and as a promising adjuvant to improve the efficacy of immunotherapeutic strategies in breast cancer.

## 10. Discussion

Breast cancer remains one of the leading causes of cancer-related death among women worldwide. Over the last two decades, its therapy has undergone a remarkable transformation with the advent of molecular target therapies, including endocrine therapy, anti-HER2 agents, PARP1 inhibitors, immune checkpoint inhibitors and the ADCs. All these strategies have significantly improved the prognosis of patients with target-addicted breast cancers. Growing evidence has demonstrated that targeting DDR is a promising therapeutic strategy in BC therapy, especially in tumors harboring alterations in DDR pathways. In this context, one of the most important recent advances for TNBC, the most aggressive subtype of BC, carrying the HRD, has been the use of Olaparib, where synthetic lethality selectively induces tumor cell death, sparing normal cells. However, one of the major challenges associated with targeted therapies is the emergence of resistance mechanisms, which negatively affect overall patient survival. Therefore, the discovery of new molecules useful both for conventional cancer therapies and for overcoming acquired resistance mechanisms is urgent. Historically, natural extracts have represented one of the main sources of potentially useful molecules for cancer therapy [133]. However, natural compounds should no longer be regarded as simple cytotoxic agents; rather, they display multifunctional abilities to influence several molecular pathways involved in tumor progression. The studies discussed in this review demonstrated that natural compounds interfere with cancer cell biology through different mechanisms, including modulation of oxidative stress, replication stress, checkpoint signaling, homologous recombination deficiency, apoptosis, and metabolic rewiring (Table 1).

**Figure 8:**
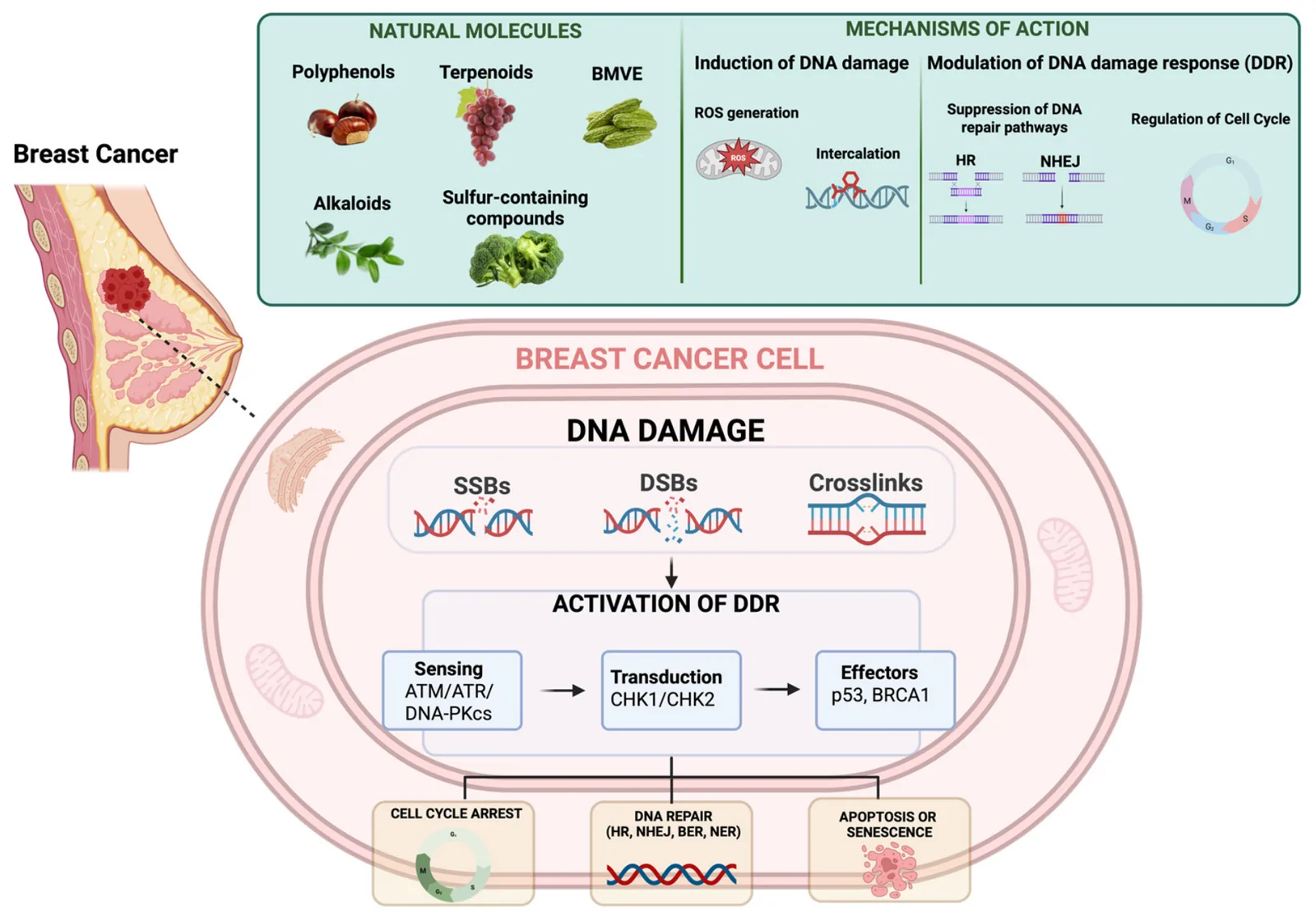
Natural compounds and DDR in breast cancer. Natural compounds from different chemical classes, including polyphenols, terpenoids, alkaloids, sulfur-containing compounds, and emerging plant-derived extracellular vesicles, exert antitumor activity in breast cancer through multiple and interconnected mechanisms. As summarized in the figure, these compounds may directly promote DNA damage by inducing reactive oxygen species (ROS) generation, replication stress, and DNA intercalation, leading to the accumulation of different DNA damage, such as single-strand breaks (SSBs), double-strand breaks (DSBs), and DNA crosslinks. Several natural compounds can instead modulate DDR signaling by altering the choice of DNA repair pathways, cell-cycle progression and sensitizing tumor cells to conventional therapies. The resulting activation of the DNA damage response, upon treatment with those compounds, engages sensing, transduction and effector pathways, including ATM/ATR/DNA-PKcs, CHK1/CHK2, p53, and BRCA1. Collectively, natural compounds emerge not only as direct cytotoxic agents but also as promising DDR modulators and chemosensitizers, supporting their potential integration into personalized therapeutic strategies for breast cancer. Created in BioRender. De Laurentiis, M. (2026) https://BioRender.com/1x1rkki.

Apigenin, castalin, selaginellins, oleanolic acid, *ent*-abietane diterpenoids and plant-derived extracellular vesicles primarily interfere with genome integrity by inducing replication stress, increasing ROS-mediated DNA damage, activating ATM signaling and influencing DNA repair pathway choice by promoting more mutagenic DNA repair pathways, resulting in the accumulation of ulterior DNA instability. Interestingly, some compounds appear to preferentially affect cell viability in TNBC models, suggesting that these tumors may be highly dependent on compensatory mechanisms required to tolerate the replication stress induced by natural compounds. In this context, natural extracts may exploit a “BRCAness-like” phenotype potentially extending the benefit of DDR-targeted therapies even in the absence of mutations in BRCA or related genes. On the other hand, several natural compounds exert their antitumor activity mainly through the modulation of oncogenic signaling pathways (Figure 8). Luteolin, for example, when combined with indole-3-carbinol, suppresses ERα signaling, while in combination with curcumin, preferentially targets TNBC, exerting an immunomodulatory activity. Similarly, cyclovirobuxine D inhibits the YAP/TAZ pathway, a key regulator of tumor growth, stemness, and therapeutic resistance. An emerging aspect is the ability of some products to act as chemosensitizers rather than simple cytotoxic agents. Among these, apigenin, castalin, oleanolic acid, piperine, and diallyl Trisulfide have been shown to enhance the efficacy of conventional chemotherapy, radiotherapy, or DDR inhibition by modulating DNA repair pathways, including HR and ATR/CHK1 signaling, as well as survival pathways. Another important consideration is the multitarget activity of natural compounds, which regulate DDR pathways, mitochondrial function, inflammatory signaling, and cancer stemness. This pleiotropic activity may represent an important advantage since acquired resistance to therapy often arises through multiple compensatory mechanisms. Apigenin represents one of the best examples of this multitarget activity by inducing ATM-dependent DDR activation, promoting cell-cycle arrest, and enhancing Doxorubicin efficacy. Piperine offers another example, not only inhibiting the PI3K/AKT/mTOR signaling and targeting breast cancer stem cells but also acting as a sensitizer of tumor cells to chemotherapy while reducing doxorubicin-induced cardiotoxicity. Likewise, erucin and diallyl trisulfide improve the efficacy of conventional chemotherapy through distinct mechanisms involving apoptosis, autophagy, antioxidant responses, or metabolic rewiring. Therefore, the simultaneous targeting of multiple pathways may increase the effectiveness of cancer treatment and reduce the likelihood of therapeutic resistance, making multitarget compounds those with the greatest preclinical efficacy. Despite these promising findings, there are several limitations that reduce the clinical translational power of these natural compounds, such as reduced solubility, rapid metabolism and pharmacokinetic variability. The advent of nanotechnology can potentially bypass these limitations by using a liposome, nanocarriers or hydrogels, increasing stability, bioavailability, tumor selectivity, and therapeutic efficacy of these natural compounds. In this context, plant-derived extracellular vesicles deserve particular attention since they offer an optimal combination of intrinsic biological activity with excellent biocompatibility and low systemic toxicity. Beyond inducing ROS-mediated DNA damage, PGEVs directly target the TME by promoting antitumor immune response, making them a promising candidate for combination with ICBs for TNBC. However, in addition to poor solubility and pharmacokinetic limitations, the clinical translation of natural compounds is also affected by the lack of standardized extraction protocols and batch-to-batch variability. All the studies reviewed here also come with a lack of deeper in vivo investigations. Future research should focus on investigating natural compounds in combination therapies according to their main activity and supported by specific molecular biomarkers such as BRCAness, DDR profiling, activation or suppression of specific oncogenic signaling pathways. In these prospective studies, natural compounds can be considered not only as single cytotoxic agents but also as adjuvant agents in combinational therapies with conventional chemotherapeutics, capable of enhancing treatment efficacy and overcoming therapeutic resistance.

## 11. Conclusions

The medical treatment of cancer has evolved significantly since the beginning of modern antitumor drug research and natural products, and their derivatives serve as essential components for treating breast cancer. Research studies demonstrate that compounds extracted from nature possess inherent cancer cell-killing abilities that improve treatment results when combined with standard cancer therapies to fight chemotherapy resistance. Many of these agents are currently in medical practice or undergoing clinical testing. Nowadays, nature continues to provide endless natural compounds to fight cancer; these compounds can act either through classical cytotoxic moieties targeting nonspecific macromolecules expressed by cancer cells and, to a lesser extent, by normal proliferating cells (e.g., DNA, enzymes, microtubules) or new compounds targeting macromolecules specifically expressed on cancer cells (e.g., oncogenic signal transduction pathways). In conclusion, the natural compounds described in this review represent the latest various substances recently investigated for their anticancer potential. Finally, the identification and characterization of novel molecules from natural sources offer a promising outlook, driving the scientific community a step closer to the goal of significantly reducing breast cancer-related mortality and improving the prognosis and quality of life for patients worldwide.

## Figures and Tables

**Table 1:** Natural products with reported activity in breast cancer models: chemical classification, sources, experimental models, and mechanisms of action along the DNA damage response (DDR) and oncogenic signaling axes. Abbreviations: DOX, Doxorubicin; ROS, reactive oxygen species; ER estrogen receptor; TNBC, triple-negative breast cancer; n.r., not reported; I3C, indole-3-carbinol; HR, homologous recombination; NHEJ, non-homologous end-joining; DSB, double-strand break; SSA, single-strand annealing; CSC, cancer stem cells; EPNE, PTX, paclitaxel; TME, tumor microenvironment; PGEvs, plant-derived extracellular vesicles (PEVs) from *P. grandiflorum*; BMVEs, bitter melon-derived vesicle-like nanostructures.

Compound	Chemical Class	Main Natural Source	Breast Cancer Models	Reported IC_50_	Effect on DDR	Effect on Oncogenic Signaling	Combination (Outcome)	Axis	Ref.
Apigenin	Flavone	Celery, parsley, chamomile, oregano; various fruits and vegetables	MDA-MB-231; MCF-7 (incl. DOX-resistant)	MCF (incl. DOX-resistant): 15 μM MDA-MD-231: 33 μM	PKCδ-dependent activation of ATM and γH2AX; G1/S arrest; downregulation of cell-cycle and DNA repair genes; no ROS generation	Inhibition of JAK2/STAT3 phosphorylation; MDR1downregulation; phytoestrogenic ER modulation	+Doxorubicin (reversal of resistance in MCF-7)	Both	[59,60,61,62,63]
Luteolin	Flavone	Reseda luteola; oregano, thyme, celery, peppermint	MCF-7, T47D (ER+); MDA-MB-231, BT-549 (TNBC); CDX mice	MCF-7: ≈50 µM alone, 30 µM in combination	n.r.	+ I3C: cyclin D1–CDK4/6 inactivation, G1 arrest, ERα-dependent; Bcl-xL/Bax modulation. + Curcumin: type I IFN activation, TGF-β suppression, ↓c-Myc, ↓Notch1	+I3C (L30I40; ER+ only); +curcumin (L30C20; TNBC only)	Signaling	[64,65,66,67,68,69]
Castalin	Ellagitannin (hydrolysable tannin)	Castanea sativa shells; Melaleuca quinquenervia leaves	MCF-7; MDA-MB-231	MCF-7: 16.1 µg/mL MDA-MB-231: 5.2 µg/mL	ROS-mediated DNA damage; HR downregulation (ZNF280A↓) with shift toward mutagenic NHEJ; enhanced CHK1 Ser345 phosphorylation	S1PR1 downregulation	+SRA737 (CHK1 inhibition; mitotic catastrophe)	Both	[70,71,72,73]
Selaginellin	Alkynylphenol pigment (selaginellin class)	Selaginella tamariscina	MDA-MB-468; MDA-MB-231	diselaginellins B MDA-MB-468: 12.5 ± 0.2 µg/mL MDA-MB-231: 3.2 ± 0.1 µg/mL	↑CHK1 and CDC25C phosphorylation; G2/M arrest; concentration-dependent ROS generation	n.r.	None reported	DDR	[74,75,76]
Oleanolic acid	Pentacyclic triterpenoid	Olea europaea; Vitis vinifera L.; apples, bilberries	MDA-MB-231	MDA-MB-231: 9.37 µg/mL HeLa cells: >10 µg/mL	Alters DSB repair pathway choice: ↓HR, promotes mutagenic SSA; radio sensitization	n.r.	+Camptothecin (↑cell death); radiation (4 Gy) + Olaparib (↑cell death)	DDR	[81,82,83]
19-BJB; 17-hydroxy-/17-acetyljolkinolide B	ent-Abietane diterpenoid	Euphorbia fischeriana Steud.	MCF-7, ZR-75-1, MDA-MB-231 (jolkinolides); T24 bladder (19-BJB)	17-hydroxy-ljolkinolide B: MCF-7: 4.7 ± 0.2 µg/mL, ZR-75-1: 2.2 ± 0.1 µg/mL MDA-MB-231: 1.1 ± 0.1 µg/mL 17-acetyl-jolkinolide B: MCF-7: 3.4 ± 0.1 µg/mL, ZR-75-1: 1.2 ± 0.1 µg/mL MDA-MB-231: 1.7 ± 0.1 µg/mL 19-BJB in T24 <6.25 µM	19-BJB: direct DNA interaction; CHK1 and CHK2 activation	n.r.	None reported	DDR	[84,85,86,87]
Cyclovirobuxine D	Steroidal (cycloartane-type) alkaloid	Buxus sinica	MCF-7, MDA-MB-231, BT-549, Hs578T; MDA-MB-231 xenograft	MCF-7: ≈40 µM MDA-MB-231: 21.7 µM BT-549: 41.9 µM Hs578T: 19.02 µM 5–10 mg/kg (in vivo)	DNA damage and impaired repair (mismatch repair; BRCA1, POLD1, BLM, MSH2, MSH6, PCNA hub—CRPC model)	Direct YAP binding: ↓YAP/TAZ nuclear translocation, ↑p-YAP; ↓CTGF, CYR61, c-Myc; FOXO3a/PINK1–Parkin mitophagy	+Doxorubicin (cardioprotection)	Both	[94,95,96,97,98]
Piperine	Piperidine amide alkaloid	Piper nigrum fruits	MDA-MB-231	Alone: 415.2 µM +DOX: 0.16 µM + PIP 100 µM	n.r.	↓p-AKT, ↓mTOR (PI3K/AKT/mTOR inhibition); ↓ALDH1 (CSC depletion)	+Doxorubicin (sensitization, cardioprotection)	Signaling	[99,100,101,102]
Erucin	Isothiocyanate (glucosinolate hydrolysis product)	Eruca sativa	T47D, MDA-MB-231	MDA-MB-231and T47D: 30 µM	n.r.	Apoptosis (caspase-3, PARP1 cleavage); autophagy (ULK1, ATG13, BECN1, BNIP3; ↑LC3-II, ↓p62); ↓ROS via H_2_S release; ↑HMOX-1, GCLC, GCLM, SOD1	+Paclitaxel (EPNE nanoemulsion; PTX-resistant T47D)	Signaling	[110,111,112,113,114,115,116,117,118,119]
Diallyl trisulfide	Allyl polysulfide (organosulfur)	Allium sativum	MCF-7, MDA-MB-231; orthotopic NOD/SCID	MCF-7: ≈125 µM MDA-MB-231 ≈ 100 µM	n.r.	Metabolic rewiring: ↓glucose uptake, ↓GLUT1, ↓LDHA, ↓HIF-1α, ↑PDH	+Doxorubicin (chemosensitization; ↓tumor volume, ↑survival)	Signaling	[120,121,122,123]
PGEVs	Plant-derived extracellular vesicles	Platycodon grandiflorum (Campanulaceae)	4T1; TNBC mouse model	4T1: 100 µg/mL	↑ROS; oxidative stress-mediated cytotoxicity and apoptosis	TME modulation: ↓PD-1, ↑CTL activity, ↑TNF-α/IL-6/IFN-γ; ↓CD31 (angiogenesis)	None reported	Both	[125,126,127,128]
BMVEs	Plant-derived vesicle-like nanostructures	Bitter melon (Momordica charantia)	4T1, MCF-7, MCF10A; 4T1-bearing mice	4T1: 20 µg/mL	ROS-mediated DNA damage	Anti-migratory activity	None reported	Both	[129,130,131,132]

## Data Availability

No new data were created or analyzed in this study. Data sharing is not applicable to this article.

## Abbreviations

The following abbreviations are used in this manuscript:

19-BJB	19-(Benzyloxy)-19-oxojolkinolide B
ADCs	Antibody–drug conjugates
AKT	Protein kinase B
ALDH1	Aldehyde dehydrogenase 1
APN	Apigenin
ATRi	ATR inhibitors
BC	Breast Cancer
BMVEs	Bitter Melon-derived Vesicle-like nanostructures
CDK4/6	Cyclin-dependent kinase 4/6
CHK1	Checkpoint kinase 1
CHK2	Checkpoint kinase 2
CPT	Camptothecin
CSC	Cancer stem cells
CVB-D	Cyclovirobuxine D
DATS	Diallyl Trisulfide
DDR	DNA damage response
DMBA	7,12-Dimethylbenz(α)anthracene
DOX	Doxorubicin
DSBs	Double-stranded DNA breaks
EAC	Ehrlich ascites carcinoma
EMA	European Medicines Agency
EPNE	Nanoemulsion of a paclitaxel and erucin combination
ER	Estrogen receptor
ER+	Estrogen receptor-positive
ESR1	Estrogen receptor 1
FDA	Food and Drug Administration
GCLC	Glutamate–Cysteine Ligase Catalytic Subunit
GCLM	Glutamate–Cysteine Ligase Modifier Subunit
GLUT1	Glucose transporter 1
GSH	Reduced glutathione
GSSG	Oxidized glutathione
HER2	Human epidermal growth factor receptor 2
HIF-1α	Hypoxia-inducible factor-1 alpha
HMOX-1	Antioxidant enzymes, including heme oxygenase-1
HR	Homologous recombination
HR+	Hormone receptor positive
HRD	Homologous recombination deficiency
I3C	Indole-3-carbinol
IC_50_	Half-maximal inhibitory concentration
ICB	Immune checkpoint blockade
IFN	Interferon
LDHA	Lactate dehydrogenase A
MMC	Mitomycin C
mTOR	Mammalian target of rapamycin
NHEJ	Non-homologous end-joining
OA	Oleanolic acid
OS	Overall survival
PARPi	Directory of open access journals
PD	Platycodin D
PD-1	Programmed death protein-1
PD-L1	Programmed death ligand-1
PDH	Pyruvate dehydrogenase
PEVEs	Plant-derived Extracellular Vesicles
PFS	Progression-free survival
PGEVs	Plant-derived Extracellular Vesicles from *P. grandiflorum*
PI3K	Phosphoinositide 3-kinase
PIP	Piperine
PPAR-γ	Peroxisome proliferator-activated receptor γ
PR	Progesterone receptor
pRb	Retinoblastoma protein
ROS	Reactive oxygen species
SERDs	Selective estrogen receptor degraders
SERMs	Selective estrogen receptor modulators
SG	Sacituzumab govitecan
SOD1	Superoxide Dismutase 1
SSA	Single-strand annealing
SSBs	Single-stranded DNA breaks
T-DM1	Trastuzumab–emtansine
T-DXd	Trastuzumab–deruxtecan
TGF-β	Transforming growth factor-β
TME	Tumor microenvironment
TNBC	Triple-negative breast cancer
Trop2	Throphoblast cell surface antigene 2
VP	Verteporfin

## References

[1] Karim A.M., Eun Kwon J., Ali T., Jang J., Ullah I., Lee Y.G., Park D.W., Park J., Jeang J.W., Kang S.C. (2023). Triple-Negative Breast Cancer: Epidemiology, Molecular Mechanisms, and Modern Vaccine-Based Treatment Strategies. Biochem. Pharmacol..

[2] Kumar N., Ehsan S., Banerjee S., Perez C.F., Lhuilier I., Neuner J., Friebel-Klingner T., Fayanju O.M., Nair B., Niinuma S.A. (2024). The Unique Risk Factor Profile of Triple-Negative Breast Cancer: A Comprehensive Meta-Analysis. J. Natl. Cancer Inst..

[3] Orrantia-Borunda E., Anchondo-Nuñez P., Acuña-Aguilar L.E., Gómez-Valles F.O., Ramírez-Valdespino C.A. (2022). Subtypes of Breast Cancer. Breast Cancer.

[4] Wang J., Wu S.G. (2023). Breast Cancer: An Overview of Current Therapeutic Strategies, Challenge, and Perspectives. Breast Cancer.

[5] Xiong X., Zheng L.W., Ding Y., Chen Y.F., Cai Y.W., Wang L.P., Huang L., Liu C.C., Shao Z.M., Yu K. (2025). Da Breast Cancer: Pathogenesis and Treatments. Signal Transduct. Target. Ther..

[6] Huppert L.A., Gumusay O., Idossa D., Rugo H.S. (2023). Systemic Therapy for Hormone Receptor-Positive/Human Epidermal Growth Factor Receptor 2-Negative Early Stage and Metastatic Breast Cancer. CA Cancer J. Clin..

[7] Gennari L. (2009). Lasofoxifene, a New Selective Estrogen Receptor Modulator for the Treatment of Osteoporosis and Vaginal Atrophy. Expert Opin. Pharmacother..

[8] Furman C., Hao M.H., Prajapati S., Reynolds D., Rimkunas V., Zheng G.Z., Zhu P., Korpal M. (2019). Estrogen Receptor Covalent Antagonists: The Best Is Yet to Come. Cancer Res..

[9] Shirley M. (2024). Capivasertib: First Approval. Drugs.

[10] Zardavas D., Baselga J., Piccart M. (2013). Emerging Targeted Agents in Metastatic Breast Cancer. Nat. Rev. Clin. Oncol..

[11] Sharma M., Kumar U., Singh S. (2025). Tumor Microenvironment in Breast Cancer: Cellular Crosstalk, Pathways, and Therapeutic Insights. Mol. Biol. Rep..

[12] Harris M.A., Savas P., Virassamy B., O’Malley M.M.R., Kay J., Mueller S.N., Mackay L.K., Salgado R., Loi S. (2024). Towards Targeting the Breast Cancer Immune Microenvironment. Nat. Rev. Cancer.

[13] Stark M.C., Joubert A.M., Visagie M.H. (2023). Molecular Farming of Pembrolizumab and Nivolumab. Int. J. Mol. Sci..

[14] Shah M., Osgood C.L., Amatya A.K., Fiero M.H., Pierce W.F., Nair A., Herz J., Robertson K.J., Mixter B.D., Tang S. (2022). FDA Approval Summary: Pembrolizumab for Neoadjuvant and Adjuvant Treatment of Patients with High-Risk Early-Stage Triple-Negative Breast Cancer. Clin. Cancer Res..

[15] Schmid P., Cortes J., Pusztai L., McArthur H., Kümmel S., Bergh J., Denkert C., Park Y.H., Hui R., Harbeck N. (2020). Pembrolizumab for Early Triple-Negative Breast Cancer. N. Engl. J. Med..

[16] Bagchi S., Yuan R., Engleman E.G. (2021). Immune Checkpoint Inhibitors for the Treatment of Cancer: Clinical Impact and Mechanisms of Response and Resistance. Annu. Rev. Pathol..

[17] Zhang L., Yan Y., Gao Y., Chen Y., Yu J., Ren N., Sun L. (2024). Antibody-Drug Conjugates and Immune Checkpoint Inhibitors in Cancer Treatment: A Systematic Review and Meta-Analysis. Sci. Rep..

[18] Schmid P., Wang H.-C., Lynce F., Asselah J., Jung K.H., Ma C.X., Park Y.H., Chen S.-C., Wysocki P.J., Baird R.D. (2026). Datopotamab Deruxtecan (Dato-DXd) in Combination with Durvalumab as First-Line Treatment for Unresectable Locally Advanced or Metastatic Triple-Negative Breast Cancer: Results from Arms 7 and 8 of the Phase Ib/II BEGONIA Study. Ann. Oncol..

[19] He F., Zhang Q., Chen Y., Ge S., Xie Y., Sun R., Wu Y., Xu J. (2025). The Regulatory Role of CDK4/6 Inhibitors in Tumor Immunity and the Potential Value of Tumor Immunotherapy (Review). Int. J. Mol. Med..

[20] Li Y., Zhang H., Merkher Y., Chen L., Liu N., Leonov S., Chen Y. (2022). Recent Advances in Therapeutic Strategies for Triple-Negative Breast Cancer. J. Hematol. Oncol..

[21] Bardia A., Hurvitz S.A., Tolaney S.M., Loirat D., Punie K., Oliveira M., Brufsky A., Sardesai S.D., Kalinsky K., Zelnak A.B. (2021). Sacituzumab Govitecan in Metastatic Triple-Negative Breast Cancer. N. Engl. J. Med..

[22] Schmid P., Abraham J., Chan S., Wheatley D., Brunt A.M., Nemsadze G., Baird R.D., Park Y.H., Hall P.S., Perren T. (2020). Capivasertib Plus Paclitaxel Versus Placebo Plus Paclitaxel As First-Line Therapy for Metastatic Triple-Negative Breast Cancer: The PAKT Trial. J. Clin. Oncol..

[23] Glaviano A., Wander S.A., Baird R.D., Yap K.C.H., Lam H.Y., Toi M., Carbone D., Geoerger B., Serra V., Jones R.H. (2024). Mechanisms of Sensitivity and Resistance to CDK4/CDK6 Inhibitors in Hormone Receptor-Positive Breast Cancer Treatment. Drug Resist. Updat..

[24] Freedman R.A., Caswell-Jin J.L., Hassett M., Somerfield M.R., Giordano S.H., Optimal Adjuvant Chemotherapy and Targeted Therapy for Early Breast Cancer Guideline Expert Panel (2024). Optimal Adjuvant Chemotherapy and Targeted Therapy for Early Breast Cancer—Cyclin-Dependent Kinase 4 and 6 Inhibitors: ASCO Guideline Rapid Recommendation Update. J. Clin. Oncol..

[25] Nur Husna S.M., Tan H.T.T., Mohamud R., Dyhl-Polk A., Wong K.K. (2018). Inhibitors Targeting CDK4/6, PARP and PI3K in Breast Cancer: A Review. Ther. Adv. Med. Oncol..

[26] Helleday T., Lo J., van Gent D.C., Engelward B.P. (2007). DNA Double-Strand Break Repair: From Mechanistic Understanding to Cancer Treatment. DNA Repair.

[27] Jackson S.P., Bartek J. (2009). The DNA-Damage Response in Human Biology and Disease. Nature.

[28] Valle I., Grinda T., Antonuzzo L., Pistilli B. (2025). Antibody–Drug Conjugates in Breast Cancer: Mechanisms of Resistance and Future Therapeutic Perspectives. npj Breast Cancer.

[29] Lee J.H., Paull T.T. (2004). Direct Activation of the ATM Protein Kinase by the Mre11/Rad50/Nbs1 Complex. Science.

[30] Zhao F., Kim W., Kloeber J.A., Lou Z. (2020). DNA End Resection and Its Role in DNA Replication and DSB Repair Choice in Mammalian Cells. Exp. Mol. Med..

[31] O’Connor M.J. (2015). Targeting the DNA Damage Response in Cancer. Mol. Cell.

[32] Mersch J., Jackson M.A., Park M., Nebgen D., Peterson S.K., Singletary C., Arun B.K., Litton J.K. (2015). Cancers Associated with BRCA1 and BRCA2 Mutations Other than Breast and Ovarian. Cancer.

[33] Ashworth A. (2008). A Synthetic Lethal Therapeutic Approach: Poly(ADP) Ribose Polymerase Inhibitors for the Treatment of Cancers Deficient in DNA Double-Strand Break Repair. J. Clin. Oncol..

[34] Lynparza|European Medicines Agency (EMA). https://www.ema.europa.eu/en/medicines/human/EPAR/lynparza.

[35] Hörner M., Hartkopf A., John N., Ziegler P., Häberle L., Uhrig S., Goossens C., Amann N., Cieslik J.P., Tretschock L.M. (2026). PARP Inhibition with Olaparib and Talazoparib for HER2-Negative Advanced Breast Cancer—Results from the Prospective PRAEGNANT Registry. npj Breast Cancer.

[36] Weigelt B., Comino-Méndez I., De Bruijn I., Tian L., Meisel J.L., García-Murillas I., Fribbens C., Cutts R., Martelotto L.G., Ng C.K.Y. (2017). Diverse BRCA1 and BRCA2 Reversion Mutations in Circulating Cell-Free DNA of Therapy-Resistant Breast or Ovarian Cancer. Clin. Cancer Res..

[37] Rottenberg S., Jaspers J.E., Kersbergen A., Van Der Burg E., Nygren A.O.H., Zander S.A.L., Derksen P.W.B., De Bruin M., Zevenhoven J., Lau A. (2008). High Sensitivity of BRCA1-Deficient Mammary Tumors to the PARP Inhibitor AZD2281 Alone and in Combination with Platinum Drugs. Proc. Natl. Acad. Sci. USA.

[38] Johnson N., Johnson S.F., Yao W., Li Y.C., Choi Y.E., Bernhardy A.J., Wang Y., Capelletti M., Sarosiek K.A., Moreau L.A. (2013). Stabilization of Mutant BRCA1 Protein Confers PARP Inhibitor and Platinum Resistance. Proc. Natl. Acad. Sci. USA.

[39] Pettitt S.J., Rehman F.L., Bajrami I., Brough R., Wallberg F., Kozarewa I., Fenwick K., Assiotis I., Chen L., Campbell J. (2013). A Genetic Screen Using the PiggyBac Transposon in Haploid Cells Identifies Parp1 as a Mediator of Olaparib Toxicity. PLoS ONE.

[40] Simoneau A., Xiong R., Zou L. (2021). The Trans Cell Cycle Effects of PARP Inhibitors Underlie Their Selectivity toward BRCA1/2-Deficient Cells. Genes Dev..

[41] Caramelo O., Silva C., Caramelo F., Frutuoso C., Pinto L., Almeida-Santos T. (2022). Efficacy of Different Neoadjuvant Treatment Regimens in BRCA-Mutated Triple Negative Breast Cancer: A Systematic Review and Meta-Analysis. Hered. Cancer Clin. Pract..

[42] Ring A., Kilburn L.S., Pearson A., Moretti L., Afshari-Mehr A., Wardley A.M., Gurel B., Macpherson I.R., Riisnaes R., Baird R.D. (2023). Olaparib and Ceralasertib (AZD6738) in Patients with Triple-Negative Advanced Breast Cancer: Results from Cohort E of the PlasmaMATCH Trial (CRUK/15/010). Clin. Cancer Res..

[43] Swisher E.M., Gonzalez R.M., Taniguchi T., Garcia R.L., Walsh T., Goff B.A., Welcsh P. (2009). Methylation and Protein Expression of DNA Repair Genes: Association with Chemotherapy Exposure and Survival in Sporadic Ovarian and Peritoneal Carcinomas. Mol. Cancer.

[44] Li X., Zou L. (2024). BRCAness, DNA Gaps, and Gain and Loss of PARP Inhibitor-Induced Synthetic Lethality. J. Clin. Investig..

[45] Wu G.Y., Xiao M.Z., Hao W.C., Yang Z.S., Liu X.R., Xu D.S., Peng Z.X., Zhang L.Y. (2025). Drug Resistance in Breast Cancer: Mechanisms and Strategies for Management. Drug Resist. Updat..

[46] Gautam S., Maurya R., Vikal A., Patel P., Thakur S., Singh A., Gupta G.D., Kurmi B.D. (2025). Understanding Drug Resistance in Breast Cancer: Mechanisms and Emerging Therapeutic Strategies. Med. Drug Discov..

[47] Zhang J., Wu Y., Li Y., Li S., Liu J., Yang X., Xia G., Wang G. (2024). Natural Products and Derivatives for Breast Cancer Treatment: From Drug Discovery to Molecular Mechanism. Phytomedicine.

[48] Lin S.R., Chang C.H., Hsu C.F., Tsai M.J., Cheng H., Leong M.K., Sung P.J., Chen J.C., Weng C.F. (2020). Natural Compounds as Potential Adjuvants to Cancer Therapy: Preclinical Evidence. Br. J. Pharmacol..

[49] Zou R., Zhou Y., Lu Y., Zhao Y., Zhang N., Liu J., Zhang Y., Fu Y. (2024). Preparation, Pungency and Bioactivity Transduction of Piperine from Black Pepper (*Piper nigrum* L.): A Comprehensive Review. Food Chem..

[50] Mitra S., Dash R. (2018). Natural Products for the Management and Prevention of Breast Cancer. Evid. Based Complement. Altern. Med..

[51] Yen C., Zhao F., Yu Z., Zhu X., Li C.G. (2022). Interactions Between Natural Products and Tamoxifen in Breast Cancer: A Comprehensive Literature Review. Front. Pharmacol..

[52] Tresserra-Rimbau A., Lamuela-Raventos R.M., Moreno J.J. (2018). Polyphenols, Food and Pharma. Current Knowledge and Directions for Future Research. Biochem. Pharmacol..

[53] Cory H., Passarelli S., Szeto J., Tamez M., Mattei J. (2018). The Role of Polyphenols in Human Health and Food Systems: A Mini-Review. Front. Nutr..

[54] Pérez-Jiménez J., Neveu V., Vos F., Scalbert A. (2010). Identification of the 100 Richest Dietary Sources of Polyphenols: An Application of the Phenol-Explorer Database. Eur. J. Clin. Nutr..

[55] Rudrapal M., Khairnar S.J., Khan J., Dukhyil A.B., Ansari M.A., Alomary M.N., Alshabrmi F.M., Palai S., Deb P.K., Devi R. (2022). Dietary Polyphenols and Their Role in Oxidative Stress-Induced Human Diseases: Insights Into Protective Effects, Antioxidant Potentials and Mechanism(s) of Action. Front. Pharmacol..

[56] Bensalem J., Dal-Pan A., Gillard E., Calon F., Pallet V. (2015). Protective Effects of Berry Polyphenols against Age-Related Cognitive Impairment. Nutr. Aging.

[57] Abbas M., Saeed F., Anjum F.M., Afzaal M., Tufail T., Bashir M.S., Ishtiaq A., Hussain S., Suleria H.A.R. (2017). Natural Polyphenols: An Overview. Int. J. Food Prop..

[58] Kumar S.S., K K., Maria E., John M. (2024). Exploring the Cytoprotective Potential of Flavonoid Fraction from *Carica papaya* L. Cultivar ‘Red Lady’ Leaf: Insights into Nrf2 Activation via in-Vitro and in-Silico Approaches. S. Afr. J. Bot..

[59] Allemailem K.S., Almatroudi A., Alharbi H.O.A., AlSuhaymi N., Alsugoor M.H., Aldakheel F.M., Khan A.A., Rahmani A.H. (2024). Apigenin: A Bioflavonoid with a Promising Role in Disease Prevention and Treatment. Biomedicines.

[60] Pandita G., Mittal D., Kashyap P., Lai W.F., Kumar N., Mehra R., Adnan M., Ashraf S.A. (2025). Apigenin and Its Derivatives in Breast Cancer Prevention and Therapy: A Review on Bioavailability and Recent Developments. Phytomed. Plus.

[61] Kashyap P., Shikha D., Thakur M., Aneja A. (2022). Functionality of Apigenin as a Potent Antioxidant with Emphasis on Bioavailability, Metabolism, Action Mechanism and in Vitro and in Vivo Studies: A Review. J. Food Biochem..

[62] Maashi M.S., Al-Mualm M., Al-Awsi G.R.L., Opulencia M.J.C., Al-Gazally M.E., Abdullaev B., Abdelbasset W.K., Ansari M.J., Jalil A.T., Alsaikhan F. (2022). Apigenin Alleviates Resistance to Doxorubicin in Breast Cancer Cells by Acting on the JAK/STAT Signaling Pathway. Mol. Biol. Rep..

[63] Arango D., Parihar A., Villamena F.A., Wang L., Freitas M.A., Grotewold E., Doseff A.I. (2012). Apigenin Induces DNA Damage through the PKCδ-Dependent Activation of ATM and H2AX Causing down-Regulation of Genes Involved in Cell Cycle Control and DNA Repair. Biochem. Pharmacol..

[64] Seelinger G., Merfort I., Schempp C.M. (2008). Anti-Oxidant, Anti-Inflammatory and Anti-Allergic Activities of Luteolin. Planta Med..

[65] Bi J., Li Y., Lu L., Yin T., Mao S., Zhang R., He J., Ni X., Wang K., Bi J. (2025). Luteolin Attenuates Inflammation and Apoptosis in LPS-Induced ALI Mice by Activating the HGF/c-Met Pathway. Front. Pharmacol..

[66] Imran M., Rauf A., Abu-Izneid T., Nadeem M., Shariati M.A., Khan I.A., Imran A., Orhan I.E., Rizwan M., Atif M. (2019). Luteolin, a Flavonoid, as an Anticancer Agent: A Review. Biomed. Pharmacother..

[67] Sato Y., Sasaki N., Saito M., Endo N., Kugawa F., Ueno A. (2015). Luteolin Attenuates Doxorubicin-Induced Cytotoxicity to MCF-7 Human Breast Cancer Cells. Biol. Pharm. Bull..

[68] Wang X., Zhang L., Dai Q., Si H., Zhang L., Eltom S.E., Si H. (2021). Combined Luteolin and Indole-3-Carbinol Synergistically Constrains ERα-Positive Breast Cancer by Dual Inhibiting Estrogen Receptor Alpha and Cyclin-Dependent Kinase 4/6 Pathway in Cultured Cells and Xenograft Mice. Cancers.

[69] Wang X., Zhang L., Si H. (2024). Combining Luteolin and Curcumin Synergistically Suppresses Triple-Negative Breast Cancer by Regulating IFN and TGF-β Signaling Pathways. Biomed. Pharmacother..

[70] Žitek Makoter T., Knez Marevci M., Knez Ž. (2024). Ellagitannin Content in Extracts of the Chestnut Wood Aesculus. Molecules.

[71] D’Angelo M., Medugno A., Cuomo M., Ragosta M.C., Russo A., Mazzarotti G., Napolitano G.M., Iannuzzi C.A., Errichiello F., Frusciante L. (2025). Castalin Induces ROS Production, Leading to DNA Damage and Increasing the Activity of CHK1 Inhibitor in Cancer Cell Lines. Antioxidants.

[72] Clarke T.L., Cho H.M., Ceppi I., Gao B., Yadav T., Silveira G.G., Boon R., Martinez-Pastor B., Amoh N.Y.A., Machin B. (2025). ZNF280A Links DNA Double-Strand Break Repair to Human 22q11.2 Distal Deletion Syndrome. Nat. Cell Biol..

[73] Xu X.Q., Huang C.M., Zhang Y.F., Chen L., Cheng H., Wang J.M. (2016). S1PR1 Mediates Anti-apoptotic/Pro-proliferative Processes in Human Acute Myeloid Leukemia Cells. Mol. Med. Rep..

[74] Li W., Tang G.H., Yin S. (2021). Selaginellins from the Genus Selaginella: Isolation, Structure, Biological Activity, and Synthesis. Nat. Prod. Rep..

[75] Ren M., Li S., Gao Q., Qiao L., Cao Q., Yang Z., Chen C., Jiang Y., Wang G., Fu S. (2023). Advances in the Anti-Tumor Activity of Biflavonoids in Selaginella. Int. J. Mol. Sci..

[76] Wen J., Sha D.M., He X.Y., Tian Y.H., Ni S.C., He B., Liu Y., Yan X.J. (2024). Selaginellin Derivatives from Selaginella Tamariscina and Evaluation for Anti-Breast Cancer Activity. Phytochemistry.

[77] Guerin M.V., Regnier F., Feuillet V., Vimeux L., Weiss J.M., Bismuth G., Altan-Bonnet G., Guilbert T., Thoreau M., Finisguerra V. (2019). TGFβ Blocks IFNα/β Release and Tumor Rejection in Spontaneous Mammary Tumors. Nat. Commun..

[78] Drasar P.B., Khripach V.A. (2021). Terpenes and Terpene Derivatives.

[79] Masyita A., Mustika Sari R., Dwi Astuti A., Yasir B., Rahma Rumata N., Emran T.B., Nainu F., Simal-Gandara J. (2022). Terpenes and Terpenoids as Main Bioactive Compounds of Essential Oils, Their Roles in Human Health and Potential Application as Natural Food Preservatives. Food Chem. X.

[80] Cox-Georgian D., Ramadoss N., Dona C., Basu C. (2019). Therapeutic and Medicinal Uses of Terpenes. Medicinal Plants: From Farm to Pharmacy.

[81] Errichiello F., D’Amato M., Gambuti A., Moio L., Pastore A., AL-Hmadi H., Stornaiuolo M., Serino E., Taglialatela-Scafati O., Forino M. (2023). Oleanolic Acid: A Promising Antidiabetic Metabolite Detected in Aglianico Grape Pomace. J. Funct. Foods.

[82] Xu A.L., Xue Y.Y., Tao W.T., Wang S.Q., Xu H.Q. (2022). Oleanolic Acid Combined with Olaparib Enhances Radiosensitization in Triple Negative Breast Cancer and Hypoxia Imaging with 18F-FETNIM Micro PET/CT. Biomed. Pharmacother..

[83] Mazzarotti G., Cuomo M., Ragosta M.C., Russo A., D’Angelo M., Medugno A., Napolitano G.M., Iannuzzi C.A., Forte I.M., Camerlingo R. (2024). Oleanolic Acid Modulates DNA Damage Response to Camptothecin Increasing Cancer Cell Death. Int. J. Mol. Sci..

[84] Yan Y., Peng M.Y., Yang Y., Zhang Z.B., Zhang L.L., Tang L., Qin X.J., Cheng Y.Y., Di Y.T., Hao X.J. (2025). Highly Oxygenated Ent-Abietane Diterpenoid Lactones from Euphorbia Peplus and Their Anti-Inflammatory Activity. Bioorg. Chem..

[85] Zhu Q.F., Xu G.B., Liao S.G., Yan X.L. (2022). Ent-Abietane Diterpenoids from Euphorbia Fischeriana and Their Cytotoxic Activities. Molecules.

[86] Chen G., Ma T., Ma Y., Han C., Zhang J., Sun Y. (2022). Chemical Composition, Anti-Breast Cancer Activity and Extraction Techniques of Ent-Abietane Diterpenoids from Euphorbia Fischeriana Steud. Molecules.

[87] Wang K., Yang J.C., Jang Y.J., Chen G.Y., Zhang Y.J., Dai Y.H., Zhang D.Y., Wu Y.C. (2021). 19-(Benzyloxy)-19-Oxojolkinolide B (19-BJB), an Ent-Abietane Diterpene Diepoxide, Inhibits the Growth of Bladder Cancer T24 Cells through DNA Damage. PLoS ONE.

[88] Ferreira M.J.U. (2022). Alkaloids in Future Drug Discovery. Molecules.

[89] Netz N., Opatz T. (2015). Marine Indole Alkaloids. Mar. Drugs.

[90] Kohnen-Johannsen K.L., Kayser O. (2019). Tropane Alkaloids: Chemistry, Pharmacology, Biosynthesis and Production. Molecules.

[91] Majnooni M.B., Fakhri S., Bahrami G., Naseri M., Farzaei M.H., Echeverría J. (2021). Alkaloids as Potential Phytochemicals against SARS-CoV-2: Approaches to the Associated Pivotal Mechanisms. Evid. Based Complement. Altern. Med..

[92] Borquaye L.S., Gasu E.N., Ampomah G.B., Kyei L.K., Amarh M.A., Mensah C.N., Nartey D., Commodore M., Adomako A.K., Acheampong P. (2020). Alkaloids from Cryptolepis Sanguinolenta as Potential Inhibitors of SARS-CoV-2 Viral Proteins: An In Silico Study. BioMed Res. Int..

[93] Griffiths M.R., Strobel B.W., Hama J.R., Cedergreen N. (2021). Toxicity and Risk of Plant-Produced Alkaloids to Daphnia Magna. Environ. Sci. Eur..

[94] Su D., Gong Y., Li S., Yang J., Nian Y. (2022). Cyclovirobuxine D, a Cardiovascular Drug from Traditional Chinese Medicine, Alleviates Inflammatory and Neuropathic Pain Mainly via Inhibition of Voltage-Gated Cav3.2 Channels. Front. Pharmacol..

[95] Xue T., Chen Y., Xu J., Du W., Kong P., Zhang X. (2023). Cyclovirobuxine D Inhibits Growth and Progression of Non-small Cell Lung Cancer Cells by Suppressing the KIF11-CDC25C-CDK1-CyclinB1 G2/M Phase Transition Regulatory Network and the NFκB/JNK Signaling Pathway. Int. J. Oncol..

[96] Wang Z., Wu Z., Chen J., Li H., Wu H., Bao J., Cheng Y., Dai Y., Wang O., Dai X. (2025). Cyclovirobuxine D Inhibits Triple-Negative Breast Cancer via YAP/TAZ Suppression and Activation of the FOXO3a/PINK1-Parkin Pathway-Induced Mitophagy. Phytomedicine.

[97] Guo Q., Guo J., Yang R., Peng H., Zhao J., Li L., Peng S. (2015). Cyclovirobuxine D Attenuates Doxorubicin-Induced Cardiomyopathy by Suppression of Oxidative Damage and Mitochondrial Biogenesis Impairment. Oxid. Med. Cell. Longev..

[98] Gao K., Zhu S., Shao Q., Qi Y., Zhang C., Li X., Guo J., Wu G., Jiang H. (2023). DNA Repair Pathways-Targeted Cyclovirobuxine Inhibits Castration-Resistant Prostate Cancer Growth by Promoting Cell Apoptosis and Cycle Arrest. Transl. Oncol..

[99] Hakeem A.N., El-Kersh D.M., Hammam O., Elhosseiny A., Zaki A., Kamel K., Yasser L., Barsom M., Ahmed M., Gamal M. (2024). Piperine Enhances Doxorubicin Sensitivity in Triple-Negative Breast Cancer by Targeting the PI3K/Akt/MTOR Pathway and Cancer Stem Cells. Sci. Rep..

[100] Quijia C.R., Chorilli M. (2022). Piperine for Treating Breast Cancer: A Review of Molecular Mechanisms, Combination with Anticancer Drugs, and Nanosystems. Phytother. Res..

[101] Lohberger B., Rinner B., Stuendl N., Absenger M., Liegl-Atzwanger B., Walzer S.M., Windhager R., Leithner A. (2012). Aldehyde Dehydrogenase 1, a Potential Marker for Cancer Stem Cells in Human Sarcoma. PLoS ONE.

[102] Yan J., Xu S.C., Kong C.Y., Zhou X.Y., Bian Z.Y., Yan L., Tang Q.Z. (2019). Piperine Alleviates Doxorubicin-Induced Cardiotoxicity via Activating PPAR-γ in Mice. PPAR Res..

[103] Castro V., Carpena M., Fraga-Corral M., Lopez-Soria A., Garcia-Perez P., Barral-Martinez M., Perez-Gregorio R., Cao H., Simal-Gandara J., Prieto M.A. (2023). Sulfur-Containing Compounds from Plants. Natural Secondary Metabolites: From Nature, Through Science, to Industry.

[104] Gambari L., Grigolo B., Grassi F. (2022). Dietary Organosulfur Compounds: Emerging Players in the Regulation of Bone Homeostasis by Plant-Derived Molecules. Front. Endocrinol..

[105] Capaldi F.R., Gratão P.L., Reis A.R., Lima L.W., Azevedo R.A. (2015). Sulfur Metabolism and Stress Defense Responses in Plants. Trop. Plant Biol..

[106] Miekus N., Marszałek K., Podlacha M., Iqbal A., Puchalski C., Swiergiel A.H. (2020). Health Benefits of Plant-Derived Sulfur Compounds, Glucosinolates, and Organosulfur Compounds. Molecules.

[107] Barman H., Kabir M.E., Borah A., Afzal N.U., Loying R., Sharmah B., Ibeyaima A., Kalita J., Manna P. (2025). Therapeutic Uses of Dietary Organosulfur Compounds in Response to Viral (SARS-CoV-2)/Bacterial Infection, Inflammation, Cancer, Oxidative Stress, Cardiovascular Diseases, Obesity, and Diabetes. Chem. Biodivers..

[108] Liu P., Zhang B., Li Y., Yuan Q. (2024). Potential Mechanisms of Cancer Prevention and Treatment by Sulforaphane, a Natural Small Molecule Compound of Plant-Derived. Mol. Med..

[109] Ali U., Wabitsch M., Tews D., Colitti M. (2023). Effects of Allicin on Human Simpson-Golabi-Behmel Syndrome Cells in Mediating Browning Phenotype. Front. Endocrinol..

[110] Genah S., Ciccone V., Filippelli A., Simonis V., Martelli A., Piragine E., Pagnotta E., Pecchioni N., Calderone V., Morbidelli L. (2024). Erucin, a Natural Isothiocyanate, Exerts pro-Angiogenic Properties in Cultured Endothelial Cells and Reverts Angiogenic Impairment Induced by High Glucose. Phytother. Res..

[111] Jakubíková J., Sedlák J., Mithen R., Bao Y. (2005). Role of PI3K/Akt and MEK/ERK Signaling Pathways in Sulforaphane- and Erucin-Induced Phase II Enzymes and MRP2 Transcription, G2/M Arrest and Cell Death in Caco-2 Cells. Biochem. Pharmacol..

[112] Lamy E., Mersch-Sundermann V. (2009). MTBITC Mediates Cell Cycle Arrest and Apoptosis Induction in Human HepG2 Cells despite Its Rapid Degradation Kinetics in the in Vitro Model. Environ. Mol. Mutagen..

[113] Azarenko O., Jordan M.A., Wilson L. (2014). Erucin, the Major Isothiocyanate in Arugula (Eruca Sativa), Inhibits Proliferation of MCF7 Tumor Cells by Suppressing Microtubule Dynamics. PLoS ONE.

[114] Banu H., Shammari E.A., Qanash H., Patel M., Adnan M., Shahanawaz S., Khan M.I., Qattan M.Y., Ashraf S.A. (2026). Erucin Targets Oncogenic Signaling Pathways in Triple-Negative Breast Cancer: An Integrated Network Pharmacology and In Vitro Study. Life.

[115] Pawlik A., Słomińska-Wojewódzka M., Herman-Antosiewicz A. (2016). Sensitization of Estrogen Receptor-Positive Breast Cancer Cell Lines to 4-Hydroxytamoxifen by Isothiocyanates Present in Cruciferous Plants. Eur. J. Nutr..

[116] Prełowska M., Kaczyńska A., Herman-Antosiewicz A. (2017). 4-(Methylthio)Butyl Isothiocyanate Inhibits the Proliferation of Breast Cancer Cells with Different Receptor Status. Pharmacol. Rep..

[117] Kaur H., Kaur K., Singh A., Bedi N., Singh B., Alturki M.S., Aldawsari M.F., Almalki A.H., Haque S., Lee H.J. (2022). Frankincense Oil-Loaded Nanoemulsion Formulation of Paclitaxel and Erucin: A Synergistic Combination for Ameliorating Drug Resistance in Breast Cancer: In Vitro and in Vivo Study. Front. Pharmacol..

[118] Bello I., Smimmo M., d’Emmanuele di Villa Bianca R., Bucci M., Cirino G., Panza E., Brancaleone V. (2023). Erucin, an H2S-Releasing Isothiocyanate, Exerts Anticancer Effects in Human Triple-Negative Breast Cancer Cells Triggering Autophagy-Dependent Apoptotic Cell Death. Int. J. Mol. Sci..

[119] Predmore B.L., Lefer D.J., Gojon G. (2012). Hydrogen Sulfide in Biochemistry and Medicine. Antioxid. Redox Signal..

[120] Puccinelli M.T., Stan S.D. (2017). Dietary Bioactive Diallyl Trisulfide in Cancer Prevention and Treatment. Int. J. Mol. Sci..

[121] Lu L., Gao Z., Song J., Jin L., Liang Z. (2024). The Potential of Diallyl Trisulfide for Cancer Prevention and Treatment, with Mechanism Insights. Front. Cell Dev. Biol..

[122] Chang C.M., Wang W.J., Mhone T.G., Ho W.K., Chen C.J., Ng S.S.C., Li C.C., Kuo C.H., Huang C.Y., Kuo W.W. (2025). Diallyl Trisulfide Enhances Doxorubicin Chemosensitivity by Inhibiting the Warburg Effect and Inducing Apoptosis in Breast Cancer Cells. J. Cancer.

[123] Liu S., Zhang X., Wang W., Li X., Sun X., Zhao Y., Wang Q., Li Y., Hu F., Ren H. (2024). Metabolic Reprogramming and Therapeutic Resistance in Primary and Metastatic Breast Cancer. Mol. Cancer.

[124] Yan G., Xiao Q., Zhao J., Chen H., Xu Y., Tan M., Peng L. (2024). Brucea Javanica Derived Exosome-like Nanovesicles Deliver MiRNAs for Cancer Therapy. J. Control. Release.

[125] Sheng Y., Liu G., Wang M., Lv Z., Du P. (2017). A Selenium Polysaccharide from Platycodon Grandiflorum Rescues PC12 Cell Death Caused by H2O2 via Inhibiting Oxidative Stress. Int. J. Biol. Macromol..

[126] Xu C., Sun G., Yuan G., Wang R., Sun X. (2014). Effects of Platycodin D on Proliferation, Apoptosis and PI3K/Akt Signal Pathway of Human Glioma U251 Cells. Molecules.

[127] Seo Y.S., Kang O.H., Kong R., Zhou T., Kim S.A., Ryu S., Kim H.R., Kwon D.Y. (2018). Polygalacin D Induces Apoptosis and Cell Cycle Arrest via the PI3K/Akt Pathway in Non-Small Cell Lung Cancer. Oncol. Rep..

[128] Yang M., Guo J., Li J., Wang S., Sun Y., Liu Y., Peng Y. (2025). Platycodon Grandiflorum-Derived Extracellular Vesicles Suppress Triple-Negative Breast Cancer Growth by Reversing the Immunosuppressive Tumor Microenvironment and Modulating the Gut Microbiota. J. Nanobiotechnol..

[129] Dandawate P.R., Subramaniam D., Padhye S.B., Anant S. (2016). Bitter Melon: A Panacea for Inflammation and Cancer. Chin. J. Nat. Med..

[130] Sur S., Ray R.B. (2021). Diverse Roles of Bitter Melon (*Momordica charantia*) in Prevention of Oral Cancer. J. Cancer Metastasis Treat..

[131] Rajamoorthi A., Shrivastava S., Steele R., Nerurkar P., Gonzalez J.G., Crawford S., Varvares M., Ray R.B. (2013). Bitter Melon Reduces Head and Neck Squamous Cell Carcinoma Growth by Targeting C-Met Signaling. PLoS ONE.

[132] Feng T., Wan Y., Dai B., Liu Y. (2023). Anticancer Activity of Bitter Melon-Derived Vesicles Extract against Breast Cancer. Cells.

[133] Popovici V., Ozon E.A., Gîrd C.E., Lupuliasa D. (2026). Advances in Plant-Sourced Natural Compounds as Anticancer Agents. Molecules.

